# GWAS-based identification of multi-trait genetic loci conferring salinity tolerance in barley under hydro- and nanoparticle-priming conditions

**DOI:** 10.1186/s12870-025-07898-5

**Published:** 2025-12-23

**Authors:** Wesam W. Abozaid, Samar G. Thabet, Mohamed A. Karam, Yasser S. Moursi

**Affiliations:** Department of Botany, Faculty of Science, University of Fayoum, Fayoum, 63514 Egypt

**Keywords:** Salt stress, Barley, Hydro priming, Zinc oxide nanoparticles, Pleiotropic effect, GWAS

## Abstract

**Background:**

Salinity is one of the most detrimental abiotic stressors, harming agricultural plants and reducing production. Barley (*Hordeum vulgare* L.) is the fourth most produced cereal crop in the world, which tolerates salt stress conditions. However, the genetic basis of salinity tolerance under hydro and nano priming in barley remains poorly understood. Therefore, understanding the genetics of seed germination under salt stress can aid in improving the development and production of barley. This study aimed to detect the genetic associations underpinning the impact of hydro and zinc oxide nanoparticles (100 ppm) priming compared to unprimed conditions on 170 spring barley cultivars that were exposed to 200 mM sodium chloride during seed germination and seedling growth using GWAS (MLM + K, *p-*value ≤ 0.0001 and FDR α = 0.01).

**Results:**

High phenotypic variations were detected among all genotypes under control and salinity for all studied traits under all treatments. The reduction rate in root length and shoot length was lower in nano and hydro priming compared to unprimed conditions. The reduction rates were higher in the nano priming compared to hydropriming and unprimed conditions for fresh weight. GWAS analysis reveals 137 and 79 significant SNPs at *p* ≤ 0.0001 and FDR ≤ 0.01, respectively. Thirty pleiotropic markers linked with salt tolerance at *p* ≤ 0.0001 for unprimed and priming conditions. For instance, on chr 7 H at position 111,292,841 bp, the SNP marker BOPA1_1518 − 624 was significantly associated with twelve traits related to germination under hydro and nano priming was found near the gene *HORVU.MOREX.r3.7HG0670060* coding for F-box family protein (protein turnover). Also, five SNPs were detected that control seven germination-related traits under all treatments. For seedling-related traits, the SNP marker SCRI_RS_168580 on 4 H was found to be associated with the salt tolerance index of both root and shoot length under unprimed conditions, which lies near the gene model *HORVU.MOREX.r3.4HG0345880* coding for peroxidase (ROS detoxification).

**Conclusions:**

Hydro and nano priming significantly enhanced seed germination and the seedling establishment traits under salinity. This study highlights the potential of employing pleiotropic markers linked to multiple traits to advance our understanding and improvement of salinity tolerance in barley.

**Supplementary Information:**

The online version contains supplementary material available at 10.1186/s12870-025-07898-5.

## Background

Salinity is a major abiotic stress that severely limits barley growth, development, and yield [1]. It also represents one of the most critical environmental challenges constraining agricultural productivity worldwide [2, 3]. Salt stress causes the accumulation of sodium (Na⁺) and chloride (Cl⁻) ions in plant tissues [4]. Excessive levels of these ions are toxic, disrupting cellular functions, enzyme activities, and metabolic processes [5, 6]. Salinity further induces oxidative stress by increasing the production of reactive oxygen species (ROS). If not neutralized by the antioxidant defense system, ROS can cause cellular damage, lipid peroxidation, protein degradation, and DNA mutations [7]. Gaining insight into the effects of salinity on barley is essential for formulating effective management strategies [6, 8]. High soil salinity significantly reduces the rate and uniformity of barley seed germination [9]. Barley (*Hordeum vulgare L*.), the fourth most widely cultivated cereal crop worldwide, is used for food, livestock feed, and the brewing industries [4, 10]. It is also the most salt-tolerant among cereals, while bread wheat shows only moderate resistance [11]. The high genetic variability of barley in stress tolerance makes it a suitable model crop for studying the genetics of developmental and adaptive traits [12]. Salinity tolerance is a complex physiological and genetic trait regulated by multiple quantitative trait loci (QTLs) [13]. Marker assisted breeding can greatly benefit from identifying QTLs underlying tolerance [14]. Earlier studies have used physiological and agronomic traits as selection criteria to detect QTLs associated with salt tolerance in barley [15, 16]. For example, Thabet et al. (2022) [17] found QTLs linked to antioxidant components under salt stress, including a single-nucleotide polymorphism (SNP) A: C on chromosome 7 H that regulates SOD_S and APX_S expression. The same group also reported quantitative trait nucleotides (QTNs) in response to selenium nanoparticle application under mild salinity [18]. Genome-wide association studies (GWAS) provide a powerful approach to uncover genetic variation underlying complex traits, including morphological and agronomic responses to abiotic stresses. Seed priming, a widely used pre-sowing technique, enhances seed quality and germination energy [19]. It increases metabolic activities associated with seed germination and early seedling growth, and as a result, seeds that can withstand abiotic stress situations like water shortages emerge [20]. Zinc is an essential micronutrient for all living things, and the presence of this element is necessary for many enzymes to operate properly [21]. The use of nanotechnology has been on the increase in varied fields, including agriculture [22]. Nanoparticles act as sources of micronutrients, supplying plants with essential nutrients, and enhancing their resistance to various kinds of stress [23]. The seed priming technique has been implemented in crops to enhance the seed quality, which is essential for strong stand establishment and improved crop productivity, even under unfavorable environmental conditions [24]. Common methods include hydropriming (water), Osmo priming (polyethylene glycol or salts), hormonal priming, and nutrient priming [24]. Nanoparticles influence nutrient absorption and chemical processes in plants, leading to improved growth and higher crop yields [25]. Despite advances in QTL mapping and GWAS for salinity tolerance, the genetic basis of tolerance under hydro and -nano priming remains poorly understood. Conventional salinity of GWAS studies typically identify loci associated with direct stress responses. However, seed priming modifies- early physiological and metabolic processes, activating antioxidant pathways, enhancing nutrient uptake, and improving germination vigor. Conducting GWAS under priming conditions, therefore, enables the discovery of genetic loci specifically linked to priming-mediated tolerance mechanisms, rather than general salinity tolerance alone. This provides novel insights into genotype × treatment interactions and highlights alleles that may be particularly valuable in breeding programs where seed priming is applied as a practical management strategy. Therefore, this study aims to: (1) characterize natural phenotypic and genetic variation in seed germination and seedling traits under salt stress in unprimed, hydropriming, and nano priming conditions across 170 spring barley accessions; and (2) identify genetic loci associated with germination and seedling growth under salt stress using GWAS in hydro and nano primed barley accessions.

## Materials and methods

### Plant material

A set panel of 170 worldwide spring barley accessions representing diverse geographical origins was used in the current study. Detailed information on each accession, including biological status (landrace, modern cultivar, wild barley) and geographical origin, is provided in Supplementary Table S1. The seeds were provided by IPK- Gene Bank Gatersleben in Germany. More information about the population structure has been published by Comadran et al., (2012) [26].

### Salt stress and nano priming concentrations

The concentrations of sodium chloride (NaCl) and zinc oxide nanoparticles were selected based on previous studies [6, 27, 28], and validated through a preliminary experiment to find the concentration that showed variation among genotypes.

### Seed priming and germination test

To compare the effects of hydro priming (H; water) and nano priming (N; ZnO nanoparticles) on seed germination and seedling establishment under control and salinity stress, zinc oxide nanoparticles (ZnO NPs; Sigma Aldrich) were used. According to the manufacturer’s specification, these ZnO NPs are high-purity ZnO nanoparticles with a nominal primary particle size of 20–30 nm, indicating a nanoscale particle-size distribution within this range. A 100 ppm ZnO NPs solution was freshly prepared by dispersing nanoparticles in deionized water using ultrasonic vibration for 20 min. For both hydro and nano priming treatments, 120 seeds were soaked in 10 ml distilled water or 100 ppm ZnO NPs solution for 12 h in darkness at 10 °C. Primed seeds were dried to their original moisture content, stored in polyethylene bags, and kept at 4 °C until further use. In addition, 120 seeds were used without priming as unprimed controls (UP). For each treatment class (unprimed, hydro primed, and nano primed), 20 seeds per genotype were germinated on two filter papers in a 9 cm sterile Petri dish, with either 10 ml distilled water or 200 mM NaCl applied immediately after priming. The experiment was conducted in a randomized complete block design with three biological replicates, each consisting of one Petri dish per genotype per treatment. Petri dishes were incubated at 20 °C in darkness, and seeds were considered germinated when the radicle extended at least 2 mm.

### Phenotyping and traits’ scoring

For up to 10 days, several germination- and seedling-related traits were calculated.

Germination was recorded at 24-hour intervals. In accordance with the International Seed Testing Association (ISTA, 2018) guidelines, germination percentage (G%), germination rate index (GRI), and germination pace (GP) were calculated at the end of the experiment (Table 1).

To measure shoot and root length, 10 seeds from each genotype were placed on rolling paper [29]. The papers were then placed in 1 L beakers containing either water (control) or a 200 mM NaCl solution (salinity treatment). The NaCl solution was refreshed every two days until the experiment concluded. After 10 days, shoot length (SL) and root length (RL) were manually recorded in centimeters. Measurements were taken on individual seedlings and averaged per replicate.

Using a delicate balance, the seedlings’ fresh weight (FW) was measured in grams.

All measured traits under all treatments, along with their names, abbreviations, and corresponding descriptions, are listed in Table 1.

**Table 1:** The name, abbreviation, and corresponding descriptions of all measured traits under all treatments

Trait	Abbreviation	Description of trait measurement
Root Length	RL	A scaled ruler was used to measure the root length (in centimeters).
Shoot Length	SL	A scaled ruler was used to measure the shoot length (in centimeters).
Fresh Weight	FW	Using a sensitive balance (Sartorius AC 1215, Germany), the fresh weight was measured in grams.
Germination Percentage	G%	(G% %)$\:=\frac{\mathrm{n}}{\mathrm{N}}$×100 where N is the total number of seeds sown, and n is the total number of seeds that germinated at the end of the experiment.
Germination Rate Index	GRI	GRI = G1/1 + G2/2+…. +Gi/i; where G1 stands for the germination % on day 1, G2 stands for the germination percentage on day 2, and so forth.
Germination Pace	GP	$GP=\frac N{\sum_{(n\times d)}}\times100$ Where n is the number of seeds that germinated on a given day, d is the day, and N is the total number of seeds that germinated at the end of the experiment.
**Salt tolerance indices (STIs)**		
Salt tolerance index (RL)	RL_STI	$STI=\frac{Trait\;value\;under\;salinity}{Trait\;value\;under\;control}\times100$
Salt tolerance index (SL)	SL_STI	
Salt tolerance index (FW)	FW_STI	
Salt tolerance index (G%)	G%_STI	
Salt tolerance index (GRI)	GRI_STI	
Salt tolerance index (GP)	GP_STI	
**Reduction (Red)**		
Root Length Reduction	RL_Red	Reduction of Trait = Trait value under control - Trait value under salinity
Shoot Length Reduction	SL_Red	
Fresh Weight Reduction	FW_Red	
Germination % Reduction	G%_Red	
Germination Rate Index Reduction	GRI_Red	
Germination Pace Reduction	GP_Red	

### Data analyses

PlabSTAT 3.0 software [30] was used to analyze the data using an analysis of variance (ANOVA) to show statistically significant changes in attributes linked to germination and growth performance under all treatments based on the following model:$Y_{ijk}=\mu+g_i+r_j+t_k+t_{ik}+tgr_{ijk}$

Where $Y_{ijk}$ is the observation of genotype i in replication j in treatment (control vs. salt stress) $k$, µ is the general mean; $g_i$, $r_j$, $t_k$ are the main effects of genotypes, replications, and treatments, respectively. $t_{ik}$ is genotype × treatment interaction. $tgr_{ijk}$ is genotype × replications × treatment interaction (error).

Additionally, the following formula was used to compute broad sense heritability (*H*^*2*^) using PLABSTAT:.

$$H^2=\sigma^2g/(\sigma^2g+\sigma^2gT/T+\sigma^2e/Tr)$$

where σ²g = genotypic variance, σ²g×T = genotype × treatment interaction variance, σ²e = residual error, r = number of replicates, and e = number of treatments.

Residual Maximum Likelihood (REML) was used to examine the phenotypic data in a mixed linear model (MLM).

### Genome-wide association scan underlying the studied traits

The 170 spring barley accessions analyzed here belong to the elite cultivated germplasm characterized by Comadran et al. (2012) [26], who reported high genome-wide diversity, extended linkage disequilibrium around centromeric regions, and clear population sub-structure resolved into three main genetic clusters (two-row spring, winter, and six-row spring/Manchurian types) using PCO and STRUCTURE analyses. To account for this structure in our GWAS, we performed principal component analysis (PCA) on the filtered SNP dataset and included the first three principal components as fixed covariates, together with the kinship (K) matrix as a random effect, in the mixed linear model. In the current study, markers were filtered based on a minor allele frequency threshold of 0.05 and a missing data rate of 0.1. As a result, 5,136 markers were retained and used for GWAS. Under all conditions, association scans between SNP markers and traits were performed using a mixed linear model (MLM) implemented in the rMVP package (version 1.4.5) in R software version 4.4.2. To account for both relatedness and population structure, the MLM included a kinship (K) matrix as a random effect and the first three principal components (PCs) derived from the SNP data as fixed covariates (K + PCA model). Association signals with P-values ≤ 0.0001 were considered significant and subjected to further bioinformatic analyses. In addition, multiple testing corrections were applied using the false discovery rate (FDR) with an α level of 0.01. Linkage disequilibrium (r²) was calculated for significant SNPs found on the same chromosome using TASSEL software version 5 [31]. Manhattan and quantile–quantile plots were generated with the rMVP package in R. The GWAS procedure employed in this study followed the approach described by reference [32].

### Candidate gene associated with the studied traits

Significant SNPs were used to show candidate genes. The barley genome web-based platform (BARLEX IPK;https://apex.ipk-gatersleben.de/apex/f? *p*=284:57, accessed 10 February 2025) and the Morex V3 reference genome were used to obtain gene annotations, gene ontology (GO), and related descriptions. Candidate genes were shown within a ± 100 kb window around each significant SNP. The SNP sequences used in this study were described in detail by Comadran et al. (2012) [26].

## Results

### Phenotypic variations

**Fig. 1:**
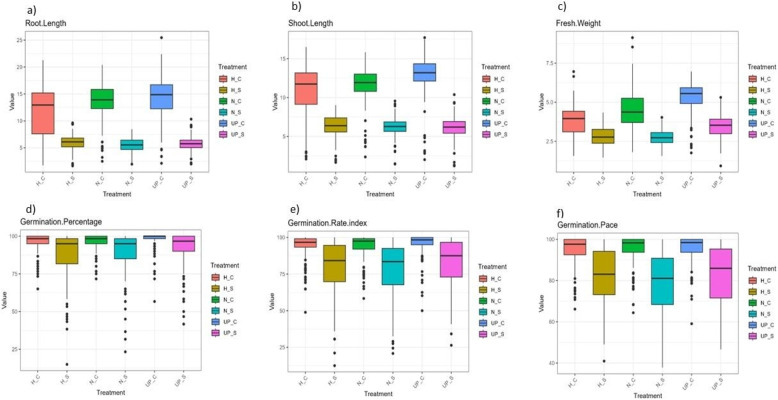
Box Plot of **a** root length (RL), **b** shoot length (SL), **c** fresh weight (FW), **d** germination percentage (G%), **e** germination rate index (GRI), and **f** germination pace (GP) under control (C) and salinity (S) for all treatments (Unprimed (UP), Hydro Priming (H), and Nano Priming (N)) in barley. H_C, H_S, N_C, N_S, UP_C, and UP_S stand for Hydro Priming control, Hydro Priming Salinity, Nano Priming Control, Nano Priming Salinity, Unprimed Control, and Unprimed Salinity, respectively

A total of 170 genotypes were evaluated for salinity tolerance at seed germination and seedling stages. Across all treatments, unprimed (UP), hydro primed (H), and nano primed (N), highly significant phenotypic differences were observed under both control (0 mM NaCl) and salinity (200 mM NaCl) conditions. Although some genotypes maintained up to 100% germination percentage (G%), germination rate index (GRI), and germination pace (GP) under salinity, overall performance was significantly reduced compared to the control (Supplementary Table S2). Root length (RL) and shoot length (SL) decreased under salinity, but reductions were less severe in N and H compared to UP (Fig. 1a–b; Supplementary Figure S2a–f). Conversely, fresh weight (FW) and GP showed greater reductions in N than in H or UP (Supplementary Figure S2g–i, p–r).

Under control conditions, RL and SL were highest in UP, while under salinity, H increased RL compared to UP and N (Fig. 1a–b and Supplementary Table S2). FW was higher in N than H under control, but the opposite was true under salinity; UP consistently showed higher FW than both priming treatments (Fig. 1c and Supplementary Table S2). For G%, N exceeded H under both conditions, though UP remained highest (Fig. 1d). Notably, 57, 32, and 39 genotypes achieved 100% G% under salinity in UP, H, and N, respectively. Similarly, 15, 5, and 6 genotypes reached 100% GRI, and 18, 8, and 7 genotypes reached 100% GP under salinity in UP, H, and N, respectively (Fig. 1e–f). Under control, N increased GRI and GP more than H, but under salinity, H outperformed N; UP consistently showed higher values than both priming treatments (Supplementary Table S2). Stress tolerance indices (STIs) and trait reductions followed normal distributions (Supplementary Figures S1–S2). The mean values under both control and salinity for all treatments are presented in Supplementary Table S2.

Analysis of variance (ANOVA) revealed high genetic variation among genotypes for all traits, with significant differences (*p* < 0.01) between control and salinity treatments. A significant genotype × treatment (G × T) interaction was detected (Tables 2, 3 and 4). Under UP conditions, G × T values ranged from 10.57** for GP to 697.77** for RL (Table 2). For H, values varied from 7.64** for GP to 125.15** for FW (Table 3), while for N they ranged from 11.27** for GP to 280.91** for SL (Table 4). These results suggest that barley responses to different environments were strongly influenced by both genetic variation and environmental factors.

**Table 2:** Analysis of variance (ANOVA) for all traits under unprimed conditions (combined ANOVA)

Sources of variance	Genotypes (G)	Treatment (T)	Replicates (*R*)	T x G	Heritability
Root Length (RL)	906.54**	638062.131**	0.59	697.77**	99.89
Shoot Length (SL)	212.10**	153892.346**	1.4	133.46**	99.53
Fresh Weight (FW)	192.30**	60463.118**	0.8	79.56**	99.48
Germination %(G%)	56.04**	1208.03**	0.07	17.27**	98.22
Germination Rate Index (GRI)	130.48**	7480.04**	0.11	40.51**	99.23
Germination Pace (GP)	22.10**	1794.57**	0.27	10.57**	95.47

*, **, *** Significant at *P* ≤ 0.05, *P* ≤ 0.01 and *P* ≤ 0.001 level of significance, respectively

**Table 3:** Analysis of variance (ANOVA) for all traits under hydro priming conditions (combined ANOVA)

Sources of variance	Genotypes (G)	Treatment (T)	Replicates (*R*)	T x G	Heritability
Root Length (RL)	58.32**	11852.18**	1.74	44.89**	98.29
Shoot Length (SL)	154.62**	28236.88**	0.04	121.40**	99.35
Fresh Weight (FW)	184.16**	16333.42**	2.1	125.15**	99.46
Germination %(G%)	60.32**	1537.65**	2.35+	19.41**	98.34
Germination Rate Index (GRI)	107.11**	5606.03**	0.78	31.61**	99.07
Germination Pace (GP)	16.82**	1523.95**	5.08**	7.64**	94.06

*, **, *** Significant at *P* ≤ 0.05, *P* ≤ 0.01 and *P* ≤ 0.001 level of significance, respectively

**Table 4:** Analysis of variance (ANOVA) for all traits under nano priming conditions (combined ANOVA)

Sources of variance	Genotypes (G)	Treatment (T)	Replicates (*R*)	T x G	Heritability
Root Length (RL)	132.57**	105060.805**	2.25	107.50**	99.25
Shoot Length (SL)	338.31**	238910.002**	0.63	280.91**	99.70
Fresh Weight (FW)	38.82**	11589.43**	1.16	31.33**	97.42
Germination %(G%)	54.37**	1256.53**	0.24	17.77**	98.16
Germination Rate Index (GRI)	120.36**	8525.34**	2.91+	37.81**	99.17
Germination Pace (GP)	22.73**	3153.01**	1.11	11.27**	95.60

*, **, *** Significant at *P* ≤ 0.05, *P* ≤ 0.01 and *P* ≤ 0.001 level of significance, respectively

Broad sense heritability (H²) estimates were consistently high, ranging from 94.06 to 99.89 across all traits (Tables 2, 3 and 4). Under UP, *H²* ranged from 95.47 for GP to 99.89 for RL (Table 2). For H, values varied from 94.06 for GP to 99.46 for FW (Table 3), and for N, they ranged from 95.60 for GP to 99.70 for SL (Table 4). The high heritability shows that these traits are predominantly controlled by genetic factors, making them desirable targets for selection in breeding programs.

### Correlation analysis

**Fig. 2:**
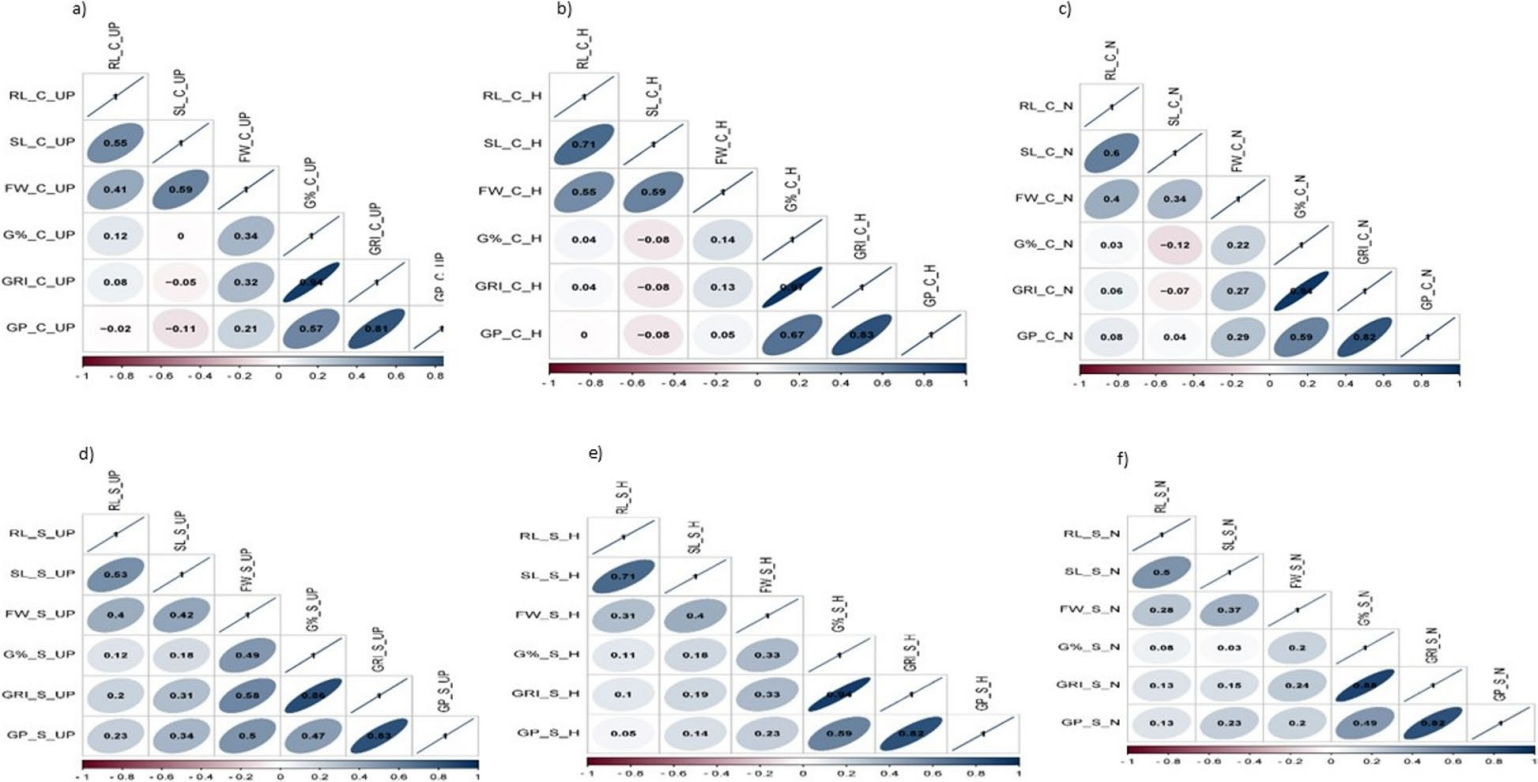
Correlation analysis of all the studied traits in barley (root length (RL), shoot length (SL), fresh weight (FW), germination percentage(G%), germination rate index (GRI), and germination pace (GP)) under control and salinity for all treatments (Unprimed (UP), Hydro Priming (H), and Nano Priming (N)): for control (C); **a** unprimed, **b** hydro priming, and **c** nano priming; for salinity (S); **d **unprimed, **e** hydro priming, and **f** nano priming

Phenotypic correlations revealed that germination traits (G%, GRI, GP) were more strongly associated with each other than with seedling traits (RL, SL, FW). Across treatments, positive correlations were generally high to very high, while negative correlations were moderate to low (Fig. 2). The strongest positive correlation was between G% and GRI, with *r* = 0.94***, 0.97***, and 0.94*** under control, and *r* = 0.86***, 0.94***, and 0.88*** under salinity for UP, H, and N, respectively.

RL showed significant positive correlations with all seedling traits under all treatments. SL correlated strongly with RL and FW under both control and salinity, with *r* = 0.80***, 0.83***, and 0.86*** for SL–RL, and *r* = 0.82***, 0.62***, and 0.64*** for SL–FW in UP, H, and N, respectively. FW showed positive correlations with all seedling traits under both control and salinity (Fig. 2).

For stress tolerance indices (STIs), the strongest correlations were between G%_STI and GRI_STI under H and N (*r* = 0.91*** and 0.85***), and between SL_STI and RL_STI under N (*r* = 0.85***). Under UP, the highest STI correlation was between SL_STI and RL_STI (*r* = 0.80***) (Supplementary Figure S3a–c). For reduction of traits, the strongest correlation was between G% and GRI (*r* = 0.76***, 0.88***, and 0.80*** for UP, H, and N, respectively) (Supplementary Figure S3d–f). Overall, germination traits showed weak correlations with seedling traits across treatments.

### GWAS mapping

**Fig. 3:**
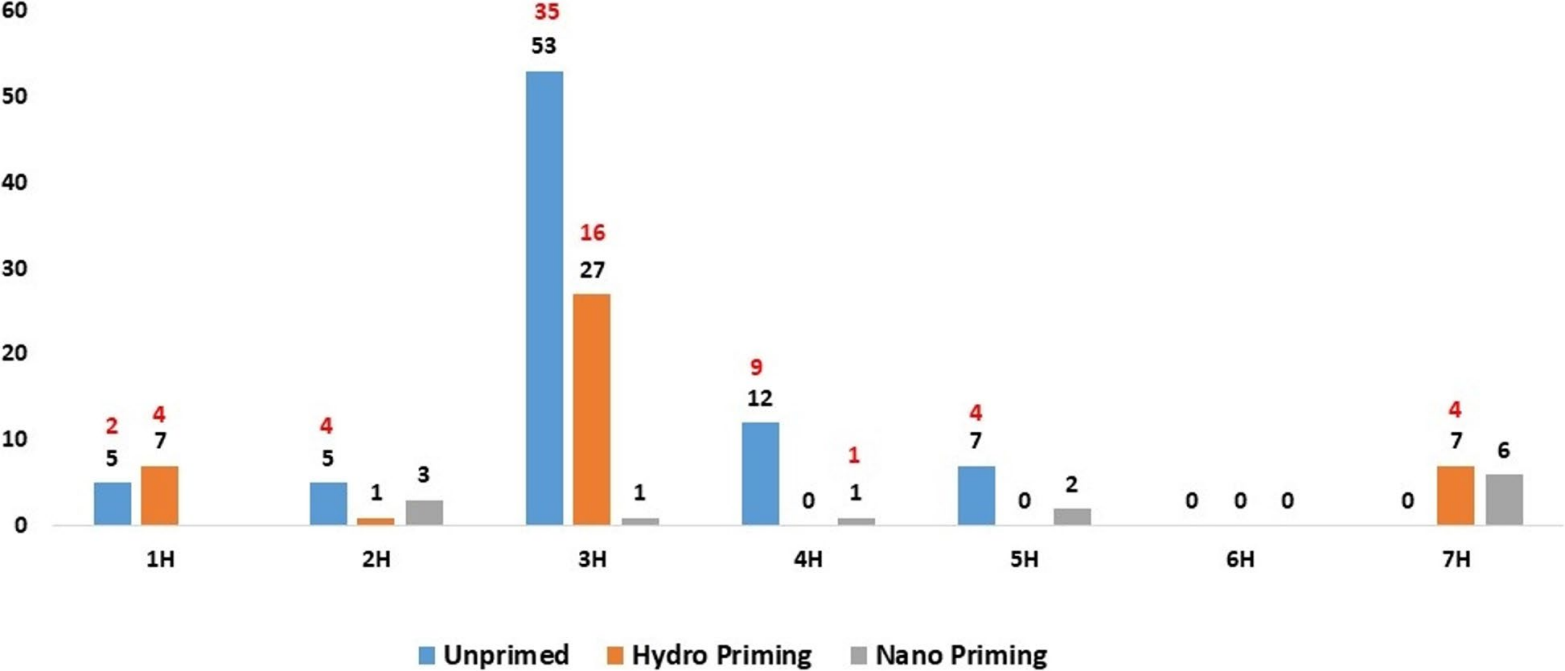
Number of SNPs per barley chromosome in each treatment (Unprimed, Hydro Priming, and Nano Priming). The black number stands for SNPs detected at *p* ≤ 0.0001, while red numbers refer to SNPs detected at *p* ≤ 0.01 FDR. The x-axis shows the chromosome number; the y-axis shows the number of SNPs

GWAS found 137 and 79 significant SNPs associated with traits across treatments at thresholds of *p* ≤ 0.0001 and FDR ≤ 0.01, respectively. The number of significant SNPs per trait and treatment is shown in Tables 5 and 6, with a full description provided in Supplementary Table S3 (-log10(p) ≥ 4). Chromosomal distribution of significant SNPs is illustrated in Supplementary Figures S4–S6, and quantile–quantile plots in Figures S7–S9. The highest number of SNPs was detected on chromosome 3 H (81 at *p* ≤ 0.0001 and 51 at FDR ≤ 0.01), while chromosome 6 H had none. Chromosomes 2 H and 5 H had nine SNPs at *p* ≤ 0.0001, while at FDR ≤ 0.01, chromosomes 2 H, 5 H, and 7 H had the lowest counts (four each) (Fig. 3). The phenotypic variation (R²) explained by individual markers ranged from 5.23% (SL_STI_UP) to 15% (FW_C_UP) (Supplementary Table S3). The highest R² was observed for FW_C_UP under UP (15%), for G%_STI_H under H (8.95%), and for RL_STI_N under N (8.71%). Notably, GWAS did not identify SNPs jointly associated with both germination-related and seedling-related traits.

**Table 5:** Number of SNPs under control, salinity, salt tolerance indices, and reduction for all treatments (unprimed conditions, hydro priming, and nano priming) at *p-*value ≤ 0.0001

	Number of SNPs				
	Control	Salinity	Salt tolerance indices	Reduction	Total SNPs
Unprimed	9	5	68	0	82
Hydro-primed	19	8	13	2	42
Nano primed	6	1	4	2	13
Total SNPs	34	14	85	4	137

**Table 6:** Summary of GWAS for all treatments under control and salinity at *p-*value ≤ 0.0001

	Traits	Treatment	Number of SNPs	chromosome	*P* value	Phenotypic Variation
Control	RL-C	Unprimed	1	5 H	7.96E-05	11.45%
		Hydro priming	-	-	-	-
		Nano priming	-	-	-	-
	SL-C	Unprimed	1	3 H	5.23E-05	5.63%
		Hydro priming	-	-	-	-
		Nano priming	-	-	-	-
	FW-C	Unprimed	1	4 H	2.94E-06	15%
		Hydro priming	1	1 H	8.36E-06	8.62%
		Nano priming	2	2 H	7.94E-05–9.28E-05	5.43–5.83%
	G%-C	Unprimed	6	1 H, 3 H, and 5 H	2.17E-05-5.42E-05	5.24–6.5%
		Hydro priming	7	1 H, 2 H, 3 H, and 7 H	7.90E-07–9.16E-05	5.89–8.2%
		Nano priming	1	3 H	3.76E-05	5.42%
	GRI-C	Unprimed	-	-	-	-
		Hydro priming	6	1 H, 3 H, and 7 H	5.47E-07-1.89E-06	7.59–8.53%
		Nano priming	2	2 H and 7 H	3.11E-05-5.57E-05	5.56–5.94%
	GP-C	Unprimed	-			
		Hydro priming	5	1 H and 3 H	3.52E-05	5.60%
		Nano priming	1	7 H	9.56E-06	6.29%
Salinity	RL-S	Unprimed	-	-	-	-
		Hydro priming	1	3 H	9.48E-05	7.39%
		Nano priming	-	-	-	-
	SL-S	Unprimed	-	-	-	-
		Hydro priming	-	-	-	-
		Nano priming	-	-	-	-
	FW-S	Unprimed	-	-	-	-
		Hydro priming	-	-	-	-
		Nano priming	-	-	-	-
	G%-S	Unprimed	5	1 H and 3 H	6.08E-05	5.23%
		Hydro priming	6	1 H, 3 H, and 7 H	2.46E-07-4.93E-06	6.89–8.87%
		Nano priming	1	7 H	7.37E-06	6.47%
	GRI-S	Unprimed	-	-	-	-
		Hydro priming	1	7 H	1.78E-05	5.99%
		Nano priming	-	-	-	-
	GP-S	Unprimed	-	-	-	-
		Hydro priming	-	-	-	-
		Nano priming	-	-	-	-
Salt Tolerance index	RL-STI	Unprimed	38	1 H, 2 H, 3 H, 4 H, and 5 H	4.07E-11-6.16E-05	5.25–14.26%
		Hydro priming	3	3 H	4.31E-06-4.12E-05	5.67–7.21%
		Nano priming	1	4 H	2.89E-07	8.71%
	SL-STI	Unprimed	18	1 H, 2 H, 3 H, 4 H, and 5 H	8.96E-09-9.39E-05	5.43–11.71%
		Hydro priming	3	3 H	5.83E-06-1.20E-05	6.35–6.81%
		Nano priming	-	-	-	-
	FW-STI	Unprimed	12	1 H, 2 H, 3 H, 4 H, and 5 H	6.35E-08-3.76E-05	5.88–10.29%
		Hydro priming	-	-	-	-
		Nano priming	2	5 H and 7 H	6.87E-05–8.89E-05	5.26–8.64%
	G%-STI	Unprimed	-	-	-	-
		Hydro priming	6	1 H, 3 H, and 7 H	1.77E-07-1.42E-06	7.62–8.95%
		Nano priming	1	7 H	8.13E-06	6.43%
	GRI-STI	Unprimed	-	-	-	-
		Hydro priming	1	7 H	1.65E-05	5.99%
		Nano priming	-	-	-	-
	GP-STI	Unprimed	-	-	-	-
		Hydro priming	-	-	-	-
		Nano priming	-	-	-	-
Reduction	RL-Red	Unprimed	-	-	-	-
		Hydro priming	-	-	-	-
		Nano priming	-	-	-	-
	SL-Red	Unprimed	-	-	-	-
		Hydro priming	-	-	-	-
		Nano priming	-	-	-	-
	FW-Red	Unprimed	-	-	-	-
		Hydro priming	1	1 H	3.30E-05	7.81%
		Nano priming	1	5 H	8.11E-05	5.34%
	G%-Red	Unprimed	-	-	-	-
		Hydro priming	1	7 H	4.03E-05	5.35%
		Nano priming	1	7 H	2.40E-05	5.71%
	GRI-Red	Unprimed	-	-	-	-
		Hydro priming	-	-	-	-
		Nano priming	-	-	-	-
	GP-Red	Unprimed	-	-	-	-
		Hydro priming	-	-	-	-
		Nano priming	-	-	-	-

### GWAS for traits under unprimed conditions (UP) at p-value ≤ 0.0001

A total of 82 significant SNPs were identified (Table 5). The phenotypic variation explained (R²) ranged from 5.23% (SL_STI_UP) to 15% (FW_C_UP) (Supplementary Table S3). Under control, the most significant SNP explained 6.5% of variation (2.17 × 10^− 5^) on chromosome 5 H with a positive allele effect of 4.16. Single SNPs were detected for RL_C_UP, SL_C_UP, and FW_C_UP on chromosomes 5 H, 3 H, and 4 H, respectively, with allele effects of 1.91 (positive), − 2.36 (negative), and 0.54 (positive) (Table 6, Supplementary Table S3).

Under salinity, significant SNPs were detected only for G%_S_UP (5 SNPs) on chromosomes 3 H and 1 H, all with *p* = 6.08 × 10^− 5^, R² = 5.23%, and negative allele effect − 26.16 (Table 6, Supplementary Table S3).

For STI traits, 68 SNPs were found. R² values ranged from 5.25 to 14.26% for RL_STI_UP, which had the highest number of SNPs (38) (Table 6 and Supplementary Table S3). The most significant SNP for RL_STI_UP was on chromosome 3 H, explaining 14.26% of variation with a positive allele effect of 66.83. The top two SNPs together accounted for 28.45% of phenotypic variation explained (PVE). For SL_STI_UP, the top two SNPs explained 22.67% of PVE with allele effects of 78.78 and 76.23. For FW_STI_UP, 12 SNPs were detected, with the most significant explaining 10.29% of variation and a positive allele effect of 31.51 (Table 6, Supplementary Table S3).

### GWAS for traits under hydro priming (H) at p-value ≤ 0.0001

Under hydropriming conditions, GWAS showed 42 SNPs across all traits (Tables 5 and 6; Supplementary Table S3). SNPs were distributed across chromosomes 1 H, 2 H, 3 H, and 7 H, with the highest number on 3 H (27 SNPs), while 2 H had only one (Fig. 3).

Under control, FW_C_H had a single SNP with a negative allele effect (–0.44). For G%_C_H, seven SNPs were detected, the most significant at *p* = 7.90 × 10^− 7^, R² = 8.2%, with a negative effect (–11.95). Six SNPs were linked to GRI, five of which had *p* = 5.47 × 10^− 7^, R² = 8.53%, and a negative effect (–23.19). GP_C_H had five SNPs at *p* = 3.52 × 10^− 5^ with a negative effect (–15.05).

Under salinity, RL_S_H had one SNP on 3 H (*p* = 9.48 × 10^− 5^, effect − 0.79). G%_S_H had six SNPs, the most significant on 7 H (R² = 8.87%, effect − 29.36). GRI_S_H had one SNP on 7 H with an effect − 29.82.

For STI traits, 13 SNPs were found (Table 5; Supplementary Figure S3). The most significant RL_STI_H SNPs were on 3 H (*p* = 4.31 × 10^− 6^, R² = 7.21%, effect + 156.06). For SL_STI_H, the top SNP had *p* = 5.83 × 10^− 6^ and effect + 94.36. For G%_STI_H, six SNPs were detected, the most significant at *p* = 1.77 × 10^− 7^ with effect − 25.9. GRI_STI_H had one SNP on 7 H with an effect of − 26.43.

For reduction of traits, FW_Red_H and G%_Red_H each had one SNP, with effects − 0.45 and + 17.28, respectively. All data are provided in Tables 5 and 6 and Supplementary Table S3.

### GWAS for traits under nano priming (N) at p-value ≤ 0.0001

Under nano priming conditions, GWAS showed 13 SNPs (Table 5). Chromosome 7 H contained the highest number (6 SNPs) (Fig. 3). Under control, single SNPs were detected for G%_C_N and GP_C_N with negative allele effects (–12.29 and − 11.12), while FW_C_N had two SNPs on 2 H, the most significant at *p* = 7.94 × 10^− 5^ with a positive effect of 0.99. GRI_C_N was associated with two SNPs, both with negative effects.

Under salinity, one SNP was detected for G%_S_N (*p* = 7.37 × 10^− 6^, effect − 23.04). For STI traits, RL_STI_N and G%_STI_N each had one SNP (*p* = 2.89 × 10^− 7^ and 8.13 × 10^− 6^) with allele effects of + 63.95 and − 20.29, respectively, while FW_STI_N had two SNPs, the most significant with a positive effect of + 12.82. For reduction of traits, FW_Red_N and G%_Red_N each had one SNP with positive effects of + 0.41 and + 16.42, respectively. A summary of GWAS results is provided in Table, with full details in Supplementary Table S3.

### SNPs with pleiotropic effect

A total of 30 pleiotropic markers were associated with at least two traits (Supplementary Table S4). Many were located near genes involved in ion transport and stress signaling, suggesting potential roles in seedling performance and germination under salt stress. Of these, seven markers were associated with germination-related traits showing both adaptive and constitutive effects, while the rest were linked to seedling traits. Notably, SNP BOPA1_1518624 on chromosome 7 H was associated with 12 traits, and five SNPs were linked to the same seven traits. In addition, 12 SNPs were associated with RL_STI_UP, SL_STI_UP, and FW_STI_UP, all with positive allele effects. The remaining pleiotropic SNPs controlled two traits.

**Fig. 4:**
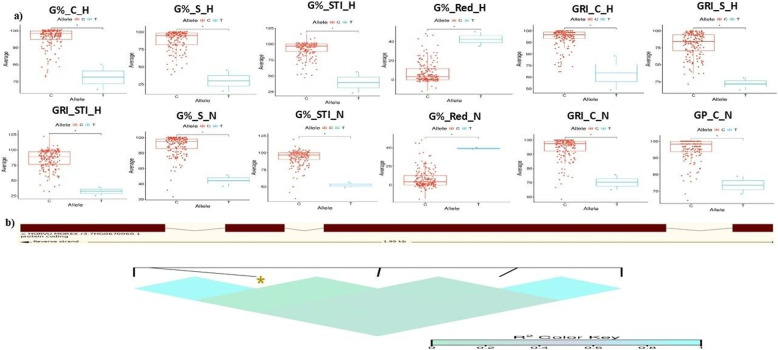
**a** Boxplot for allele effect on the twelve traits; Germination Percentage _C_H, Germination Percentage_S_H, Germination Percentage_STI_H, Germination Percentage_Red_H, Germination Rate Index_C_H, Germination Rate Index_S_H, Germination Rate Index_STI_H, Germination Percentage_S_N, Germination Percentage_STI_N, Germination Percentage_Red_N, Germination Rate Index_C_N, and Germination Pace_C_N. Where C_H, S_H, STI_H, C_N, S_N, STI_N, and Red_N stand for Hydro Priming control, Hydro Priming Salinity, Hydro Priming Salt Tolerance Index, Nano Priming Control, Nano Priming Salinity, Nano Priming_ Salt Tolerance Index, and Nano Priming_ Reduction. The x-axis shows the allele; the y-axis shows the average of traits. **b** The structure and physical position of the candidate gene at 7 H, *HORVU.MOREX.r3.7HG0670060*, the SNP BOPA1_1518 − 624 is located physically near the *HORVU.MOREX.r3.7HG0670060* with LD heatmap

**Fig. 5:**
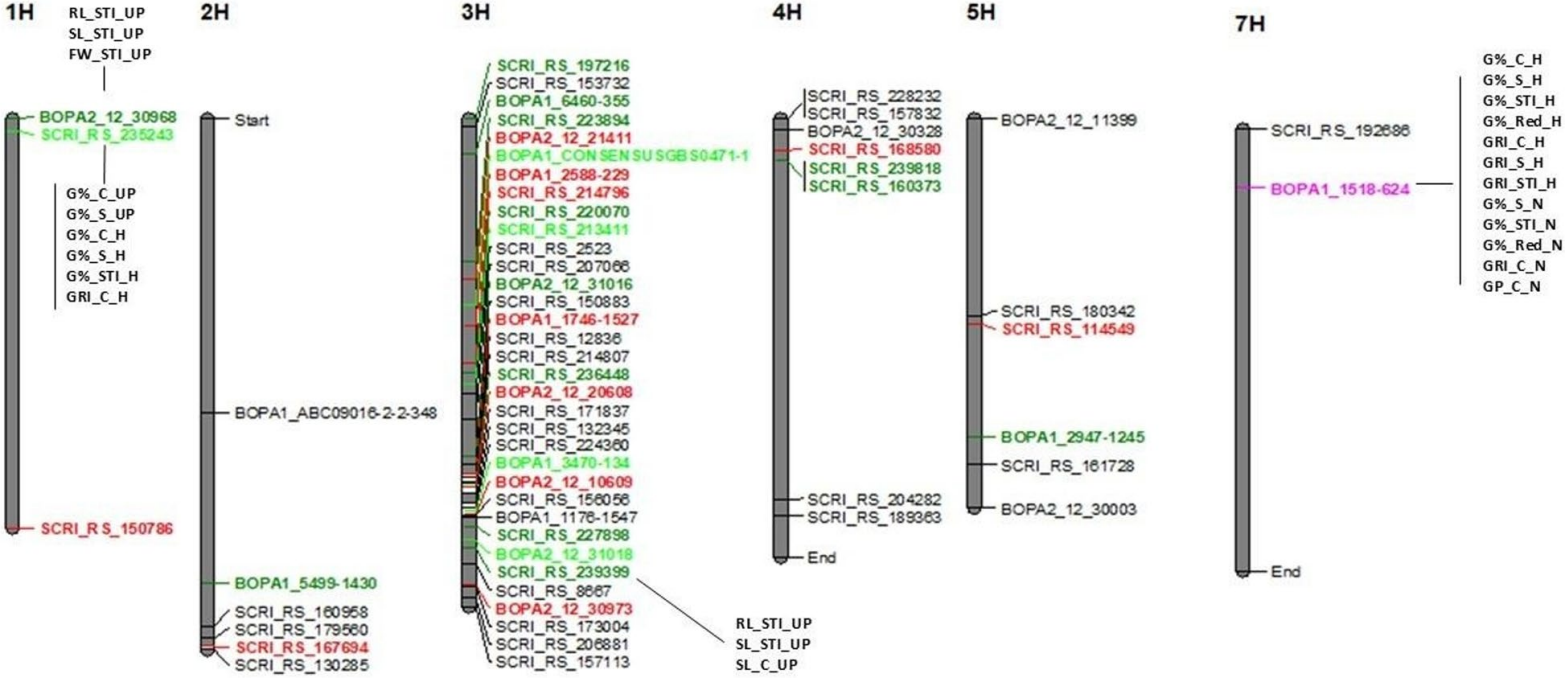
Distribution of significant SNPs on barley chromosomes in all treatments (Unprimed, Hydropriming, and Nano Priming). SNPs controlling multiple traits are highlighted in different colors: pink, light green, dark green, and red for SNPs associated with 12, 7, 3, and 2 traits, respectively. Black color for a single trait

BOPA1_1518 − 624 (chr 7 H, position 111292841 bp) was located within an LD region and significantly associated with 12 germination traits under H and N conditions (G%_C_H, G%_S_H, G%_STI_H, G%_Red_H, GRI_C_H, GRI_S_H, GRI_STI_H, G%_S_N, G%_STI_N, G%_Red_N, GRI_C_N, and GP_C_N). The allele had negative effects on all traits except G%_Red_H and G%_Red_N, which showed positive effects. Allelic analysis confirmed that the C allele significantly influenced all traits except G%_Red_H and G%_Red_N (*p* < 0.05) (Figs. 4, 5).

Five SNPs were associated with the same seven traits (G%_C_UP, G%_S_UP, G%_C_H, G%_S_H, G%_STI_H, GRI_C_H, and GP_C_H) (Supplementary Table S4). Twelve SNPs were linked to RL_STI_UP, SL_STI_UP, and FW_STI_UP; for example, SCRI_RS_227898 on chromosome 3 H was within an LD region and had positive allele effects for all three traits (Supplementary Figure S10). Another SNP, SCRI_RS_168580, was associated with RL_STI_UP and SL_STI_UP (Supplementary Table S4; Fig). Allelic analysis showed that the A allele had a highly significant effect on RL_STI_UP but a non-significant effect on SL_STI_UP (Supplementary Figure S11).

**Fig. 6:**
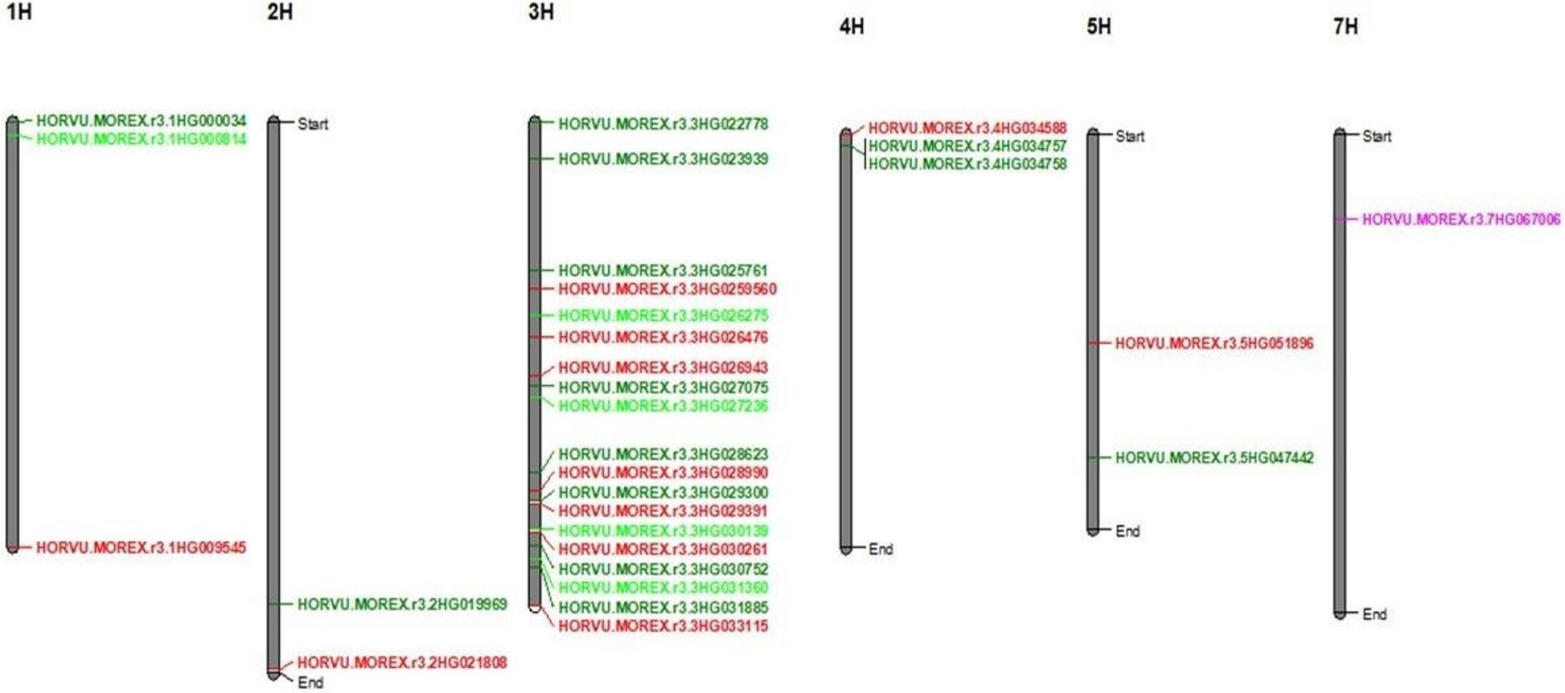
Distribution of genes on barley chromosomes in all treatments (Unprimed, Hydro Priming, and Nano Priming). Genes highlighted in different colors: pink, light green, dark green, and red for genes associated with 12, 7, 3, and 2 traits, respectively

### Gene annotation analysis

The gene annotation analysis was conducted for the pleiotropic markers (control the variation of more than one trait), and the SNPs reside within a gene model (Supplementary Table S5). Interestingly, the multi-trait associated SNPs were distributed along all chromosomes except chromosome 6 H, and two of them were detected within the gene model (Supplementary Table S5). The most important SNP (BOPA1_1518 − 624) associated with twelve germination-related traits was mapped on chromosome 7 H and showed a pleiotropic effect. It was detected near the gene model *HORVU.MOREX.r3.7HG0670060* that codes for F-box family protein (Fig. 6 and Supplementary Table S5). Moreover, this SNP was the most significant for all these traits except GRI_C_H. Interestingly, two SNPs (SCRI_RS_223894 and BOPA1_1746 − 1527) related to seedling-related traits were found near to gene *HORVU.MOREX.r3.3HG0257610* and *HORVU.MOREX.r3.3HG0289900* models, which were annotated for the NAC (No Apical Meristem) domain transcriptional regulator superfamily protein. Also, on chromosome 3 H, the pleiotropic SNP (SCRI_RS_227898) associated with RL_STI_UP, SL_STI_UP, and FW_STI_UP was detected within the gene model *HORVU.MOREX.r3.3HG0307520* that codes for bZIP transcription factor (DUF630 and DUF632) and within the LD region (Supplementary Table S5 and Supplementary Figure S10). Another SNP on chromosome 3 H was found near to gene model *HORVU.MOREX.r3.3HG0318850*, which codes for calcium-dependent protein kinase 13. On chromosome 4 H, the pleiotropic SNP SCRI_RS_168580 associated with two seedling-related traits, RL_STI_UP and SL_STI_UP, was found near the gene model *HORVU.MOREX.r3.4HG0345880*, which coded for peroxidase (Supplementary Table S5). Additionally, on chr 3 H, a pleiotropic SNP BOPA2_12_31018 associated with seven traits (G%_C_UP, G%_S_UP, G%_C_H, G%_S_H, G%_STI_H, GRI_C_H, and GP_C_H) was detected near the gene *HORVU.MOREX.r3.3HG0313600* that coding for Glutathione S-transferase (Supplementary Table S5). Furthermore, on chromosome 1 H the pleiotropic SNP (SCRI_RS_235243) associated with (G%_C_UP, G%_S_UP, G%_C_H, G%_S_H, G%_STI_H, GRI_C_H, and GP_C_H), that found near to the gene *HORVU.MOREX.r3.1HG0008140* that codes for Glycosyltransferase (Supplementary Table S5). The SNP SCRI_RS_214796 on 3 H, which is associated with RL_STI_UP and SL_STI_UP, was found within the gene *HORVU.MOREX.r3.3HG0269430* model and annotated for Chaperone protein dnaJ.

Taken together, phenotypic and genomic variations consistently identify root length-based indices as primary indicators of salinity tolerance in barley. RL_STI under unprimed and nano primed conditions exhibited the greatest variation and heritability and was associated with numerous significant SNPs, including major loci on chromosomes 3 H and 7 H. Notably, SNP BOPA1_1518 − 624 on 7 H, linked to multiple germination and seedling traits, is adjacent to an F-box protein gene (*HORVU.MOREX.r3.7HG0670060*), suggesting hormone-related signaling in priming mediated tolerance. Similarly, SNP marker (SCRI_RS_168580) on 4 H, has been mapped near to a peroxidase gene (*HORVU.MOREX.r3.4HG0345880*), this highlights the role of ROS detoxification. The lack of shared SNPs between germination traits (G%, GRI, GP) and seedling traits (RL, SL, FW), despite moderate correlations, indicates distinct genetic modules. These findings provide a framework for considering root length indices and pleiotropic loci on 3 H, 4 H, and 7 H in genomic breeding strategies for enhanced salinity tolerance.

## Discussion

### Phenotypic variation and correlation

Salinity is a major environmental stress that significantly affects barley growth, development, and yield [33]– [34]. Understanding these effects is essential for developing strategies to improve crop performance and ensure food security in saline-affected areas [17, 34]. Under salinity stress, crop plants typically exhibit reduced germination rates and slower growth [4]. Germination is a critical stage in the plant life cycle and is highly sensitive to salt stress [35]– [36]. In this study, a panel of 170 barley accessions was evaluated for salt stress tolerance during seed germination and early development under three conditions: unprimed (UP), hydro primed (H), and nano primed (N) with ZnO nanoparticles. Overall, salinity caused a significant reduction in all traits compared to control conditions, although some genotypes maintained up to 100% performance under stress.

Root and shoot length were significantly reduced under salinity compared to the control. However, the reductions were less pronounced in nano priming and hydropriming treatments than in unprimed seeds, suggesting that these priming methods partially mitigated salinity-induced growth inhibition. In contrast, fresh weight and germination pace decreased more under nano priming than under hydropriming or unprimed conditions, indicating that nano priming did not alleviate the negative effects of salinity on these traits. This result may reflect a shift in tissue composition, where nano priming promotes elongation growth but reduces tissue hydration, leading to thinner organs with lower water content rather than reduced biomass production. The absence of dry weight measurements in the present study limits our ability to confirm this interpretation; future work incorporating dry weight data will be essential to distinguish whether reduced fresh weight reflects impaired biomass accumulation or simply altered water status under nanoparticle priming. Statistical analysis revealed highly significant differences between control and salinity treatments under all conditions (UP, H, and N), confirming the strong impact of salt stress on the tested genotypes. The significant treatment × genotype (T × G) interaction showed that genotypes responded differently across traits under both treatments, highlighting the combined influence of genetic variation and environmental factors on barley performance. Similar T × G interactions under salinity have been reported in cotton [37] and sorghum [38], as well as in barley under drought stress [39].

The substantial genetic variation observed across all traits among accessions provides valuable opportunities for breeding programs aimed at developing salt-tolerant genotypes, consistent with previous findings in barley [6]. The high broad sense heritability estimates for all traits further suggest that selection will be effective for improving salt tolerance in the studied material. Our results are in agreement with earlier studies in barley [6] and wheat [40] under salinity stress. Moreover, the integration of priming techniques, particularly nano priming, appears promising for enhancing stress tolerance and merits further exploration.

Phenotypic correlation analysis revealed that germination-related traits (G%, GRI, and GP) showed stronger associations than seedling-related traits (RL, SL, and FW), indicating interdependency among germination parameters. Similar findings have been reported in barley under drought stress [41]. The strongest positive correlation was consistently observed between G% and GRI under both control and salinity conditions across all treatments (UP, H, and N) (Fig. 2). Variation in correlation strength among treatments highlights the role of priming techniques in modulating trait interactions. Hydro and nano priming enhanced positive correlations between key germination traits such as G% and GRI, suggesting their effectiveness in alleviating salinity stress. Moreover, nano priming appeared to strengthen relationships between root and shoot growth traits, pointing to its potential in improving overall seedling vigor.

The significant correlations observed emphasize the importance of germination and seedling traits in determining salinity tolerance. In particular, the consistent relationship between G% and GRI suggests that these parameters can serve as reliable indicators for screening salinity-tolerant genotypes.

### GWAS results and SNP identification

In this study, GWAS showed 137 and 79 significant SNPs across all barley chromosomes for the evaluated traits at thresholds of *p* ≤ 0.0001 and FDR ≤ 0.01, respectively (Fig. 3). The number of significant SNPs varied among treatments, with UP conditions yielding the highest count (82 SNPs), followed by H (42 SNPs) and N (13 SNPs) (Table 6; Fig. 3). At FDR ≤ 0.01, SNPs were detected for only eight traits. These results are consistent with previous reports, which suggest that salinity tolerance in barley is governed by complex interactions among multiple genes, thereby complicating the identification of associated markers [42].

Under all treatments, correlation analysis showed weak relationships between germination characteristics (G%, GRI, GP) and seedling traits (RL, SL, FW), indicating a limited functional relationship between early germination performance and later seedling vigor. Nevertheless, SNPs that were collectively linked to both sets of attributes were not found by GWAS. This combination of genetic and phenotypic data suggests that, although germination and seedling characteristics exhibit some physiological relationships, their genetic regulation seems to be mostly independent in our dataset.

The highest number of SNPs was associated with root length-related parameters, with 44 SNPs at *p* ≤ 0.0001 and 39 SNPs at FDR ≤ 0.01. This finding is consistent with previous studies in barley [41, 43–45] and underscores the critical role of roots under salinity stress, as they generate significant differences between genotypes and distinguish tolerant from susceptible lines. The results also demonstrate that the tested genotypes possess sufficient genetic diversity to be exploited in marker-assisted selection (MAS).

The phenotypic variation (R²) explained by individual markers ranged from 5.23% (SL_STI_UP) to 15% (FW_C_UP) (Supplementary Table S3). Notably, the most significant SNP for RL_STI_UP was detected on chromosome 3 H (*p* = 4.07 × 10^− 11^, R² = 14.26%), while RL_STI_N under nano priming showed the strongest association at *p* = 2.89 × 10^− 7^. These findings suggest that the identified SNPs represent major quantitative trait loci (QTLs) with substantial effects on trait variation and highlight that different genetic loci modulate priming responses to salinity in a treatment-dependent manner.

Interestingly, 30 SNPs exhibited pleiotropic effects. Pleiotropy, where a single genetic variant influences multiple phenotypes, is a well-documented phenomenon in genetics and plays a critical role in understanding trait interrelationships and biological pathways. The pleiotropic SNPs identified here have significant implications for MAS and candidate gene discovery [41, 46]. The detection of multiple pleiotropic SNPs emphasizes the complex genetic architecture underlying salinity tolerance in barley. Similar pleiotropic associations have been reported in barley under salinity [6], under drought [41], and in wheat [40, 47].

### Candidate gene functional analysis

Therefore, we focused on SNPs associated with multiple traits, prioritizing those with the strongest significance across one or more traits (Supplementary Table S4). A noteworthy finding was the pleiotropic SNP marker BOPA1_1518624, located within an LD region on chromosome 7 H (position 111292841 bp). This SNP was significantly associated with twelve germination-related traits under hydro and nano priming (G%_C_H, G%_S_H, G%_STI_H, G%_Red_H, GRI_C_H, GRI_S_H, GRI_STI_H, G%_S_N, G%_STI_N, G%_Red_N, GRI_C_N, and GP_C_N). It was the most significant for all traits except GRI_C_H. BOPA1_1518624 is located near the gene model *HORVU.MOREX.r3.7HG0670060*, which encodes an F-box family protein. The allele had negative effects on ten traits, while for G%_Red_H and G%_Red_N it showed positive effects (Supplementary Table S5).

Allele C was found to enhance germination performance while mitigating the negative effects of salinity stress (Fig. 4). This highlights its potential relevance for breeding programs aimed at improving seed vigor and stress resilience [17], although further validation across diverse populations and environments is needed. The ability of this marker to improve germination while reducing its decline under nano priming underscores its potential in improving seed treatment strategies to maximize germination success. F-box proteins are known as regulators of root growth, seed dormancy, germination, hormone signaling, and stress responses [48]– [49]. They function within the ubiquitin proteasome system, controlling protein degradation and turnover, processes essential for plant growth and stress adaptation [50]– [51]. Under salinity stress, reactive oxygen species (ROS) accumulation inhibits germination, and F-box proteins may regulate ROS scavenging enzymes, thereby enhancing stress tolerance [48]. In *Arabidopsis*, the F-box gene *AtPP2B11* was upregulated under salinity, influencing more than 4,000 genes [52]. In barley, Thabet et al. (2024) [34] identified a gene on chromosome 1 controlling APX and SOD variation under salinity with nano K application.

The significant association of SNP BOPA1_1518624 with germination stress tolerance indices (G%_STI_H, G%_STI_N, and GRI_STI_H) suggests a role in salinity tolerance via ROS regulation. Moreover, SNP trait associations observed under nano priming (G%_S_N, G%_STI_N, G%_Red_N, and GRI_C_N) further support the involvement of the *HORVU.MOREX.r3.7HG0670060* gene in mediating priming responses. The pleiotropic nature of this SNP, enhancing multiple germination traits under both H and nano priming conditions, underscores its importance for genetic improvement of barley under salinity stress.

Two SNPs were identified near genes encoding NAC domain transcriptional regulators, suggesting their involvement in barley’s response to salinity stress. The first SNP, SCRI_RS_223894 on chromosome 3 H, was linked to RL_STI_UP, SL_STI_UP, and FW_STI_UP and located near the *HORVU.MOREX.r3.3HG0257610* gene model. The second SNP, BOPA1_17461527 on 3 H, was associated with RL_STI_H and SL_STI_H and detected near *HORVU.MOREX.r3.3HG0289900*. NAC transcription factors (TFs) are among the largest plant TF families, essential for abiotic and biotic stress responses, including salinity [53]. In *Arabidopsis* and rice, NAC proteins contain a conserved N-terminal DNA-binding domain and a variable C-terminal regulatory region [54]– [55]. Thabet et al. (2024) [34] also reported a NAC-related gene on 2 H in barley under salinity with nano potassium application. The identification of NAC-associated SNPs linked to root/shoot growth, germination, and biomass maintenance highlights their potential as molecular markers for breeding salt-tolerant barley.

Notably, the pleiotropic SNP SCRI_RS_227898 on chromosome 3 H was associated with RL_STI_UP, SL_STI_UP, and FW_STI_UP under UP conditions. This SNP was found within *HORVU.MOREX.r3.3HG0307520*, which encodes a bZIP transcription factor (DUF630/DUF632). bZIP TFs play key roles in plant growth, hormone signaling, and abiotic stress responses such as salinity, drought, and osmotic stress [56, 57]. In barley, bZIP genes linked to salinity stress have been reported on chromosome 5 H [6, 58], highlighting their role in seed germination and root length regulation. In wheat, TabZIP15 enhances salt tolerance [59], while in rice, OsbZIP71 interacts with OsMyb4 to regulate salt and drought tolerance [60], and OsABF1 and OsbZIP23 are highly expressed in roots under salinity stress [61]. Despite these advances, further studies are needed to clarify bZIP interactions with other TFs and signaling pathways.

On chromosome 3 H, SNP SCRI_RS_239399 was associated with RL_STI_UP, SL_STI_UP, and SL_C_UP and was located near the *HORVU.MOREX.r3.3HG0318850* gene model encoding calcium-dependent protein kinase 13. Calcium is a crucial nutrient for plant growth and development [62]– [63] and functions as a key second messenger in stress-induced signal transduction pathways regulating gene expression [64]– [65]. Calcium-binding proteins such as Calcineurin B-like (CBL) proteins act as Ca²⁺ sensors, transmitting signals to downstream pathways via interactions with CBL-interacting protein kinases (CIPKs). These complexes regulate diverse processes, including ion homeostasis under salinity stress [66]. Understanding their precise roles across developmental stages may aid in breeding salt-tolerant barley varieties.

On chromosome 4 H, SNP SCRI_RS_168580 was associated with RL_STI_UP and SL_STI_UP and was found near *HORVU.MOREX.r3.4HG0345880*, which encodes peroxidase. Antioxidant enzymes such as catalase (CAT), superoxide dismutase (SOD), and peroxidase (POD) protect plants against oxidative stress by scavenging reactive oxygen species (ROS) [67]– [68]. Enhanced activity of these enzymes improves defense against salt stress and reduces oxidative damage [18]. Barley plants respond to salinity by strengthening antioxidant systems, including POD, SOD, and CAT, which alleviate cellular damage [7]. Peroxidases also participate in ABA-mediated signaling during salinity stress, contributing to stomatal closure and regulation of seed germination [69]– [70]. Our findings agree with previous studies reporting peroxidase-related genes in barley under selenium nanoparticles [71]. Similarly, Thabet et al. (2024) [34] found the gene *HORVU.MOREX.r3.5HG0494660* on chromosome 5 H, annotated as superoxide dismutase [Cu-Zn], in barley under salt stress with potassium nanoparticle application.

On chromosome 3 H, SNP BOPA2_12_31018 was associated with seven traits (G%_C_UP, G%_S_UP, G%_C_H, G%_S_H, G%_STI_H, GRI_C_H, and GP_C_H) and located near the *HORVU.MOREX.r3.3HG0313600* gene, which encodes Glutathione S transferase (GST). GSTs belong to a large superfamily of multifunctional enzymes that play critical roles in detoxification and oxidative stress tolerance [72]. They are involved in both biotic and abiotic stress responses [73]– [74] and are classified into cytosolic, mitochondrial, and microsomal families [75]. Induction of GSTs supports their defensive role against oxidative stress [76], and they are also regulated by phytohormones such as auxin, salicylic acid, abscisic acid, and ethylene, linking them to plant growth and development [77]. GST expression is upregulated under salt stress [78]. In *Arabidopsis*, *AtGSTU19* improved ROS stability and enhanced resistance to salinity by increasing GST and antioxidant enzyme activity [79]. Similarly, Thabet et al. (2021) [6] reported a GST gene on chromosome 5 H in barley under salt stress.

On chromosome 1 H, SNP SCRI_RS_235243 was associated with seven traits (G%_C_UP, G%_S_UP, G%_C_H, G%_S_H, G%_STI_H, GRI_C_H, and GP_C_H) and located near *HORVU.MOREX.r3.1HG0008140*, which encodes Glycosyltransferase. Glycosyltransferases are a large superfamily of enzymes that catalyze the transfer of sugar moieties from activated donors to specific acceptors [80]. In *Arabidopsis*, galacturonosyltransferase proteins contribute to xylan and pectin biosynthesis in cell walls and seed testa [81]– [82]. Ecotypic expression of glycosyltransferase enhanced salt tolerance in tobacco, improving germination and growth [83]. In rice, the glycosyltransferase gene *UGT2* plays a crucial role in salt tolerance, regulated by the *OsbZIP23* transcription factor under stress conditions [84]. These findings support our results, as this gene was associated with germination percentage under both control and salinity.

### Implications for breeding

In this study, several genes were identified as modulators of seed germination and seedling establishment under priming conditions. These include unprimed and hydropriming specific gene models *HORVU.MOREX.r3.3HG0313600* and *HORVU.MOREX.r3.1HG0008140* (germination traits), and *HORVU.MOREX.r3.2HG0115300* (seedling traits under hydropriming). Meanwhile, *HORVU.MOREX.r3.7HG0670060* was associated with germination traits under both hydro and nano priming, showing pleiotropic effects. Understanding the molecular mechanisms underlying these genes provides valuable insights into seed priming strategies and genetic improvement for stress-tolerant barley. These findings highlight the relevance of identified SNPs and candidate genes for breeding programs aimed at enhancing seed vigor and salinity tolerance, though further validation across diverse populations is needed.

## Conclusions

Salinity negatively affects seed germination and seedling traits; however, some genotypes maintained up to 100% germination percentage, germination rate index, and germination pace under both control and salinity conditions. Nano priming reduced the decline in root and shoot length compared to unprimed seeds. Several pleiotropic SNPs associated with salt stress response were identified, including one near *HORVU.MOREX.r3.7HG0670060* encoding an F-box protein, which controlled 12 germination-related traits. The identified candidate genes belong to diverse functional groups such as transcription factors, antioxidant enzymes, and ion channels, indicating that barley employs multiple mechanisms to cope with salinity. These discoveries provide a strong foundation for future crop improvement through genetic breeding. Furthermore, nanotechnology offers a promising approach to enhance plant resilience to salinity, supporting sustainable agriculture and food security in salt-affected regions.

## Supplementary Information

Supplementary material 1.

Supplementary material 2.

Supplementary material 3.

Supplementary material 4.

## Data Availability

All data generated or analyzed during this study are included in this article and its supplementary information files. All materials are available through the corresponding authors upon reasonable request.
