# Supplementary material for: GWAS-based identification of multi-trait genetic loci conferring salinity tolerance in barley under hydro- and nanoparticle-priming conditions
Source: BMC Plant Biol. 2025 Dec 23;26:166. doi: 10.1186/s12870-025-07898-5 (PMC12849565; doi:10.1186/s12870-025-07898-5)
Supplement: Supplementary file 1 — Supplementary material 1. [file 12870_2025_7898_MOESM1_ESM.pdf]

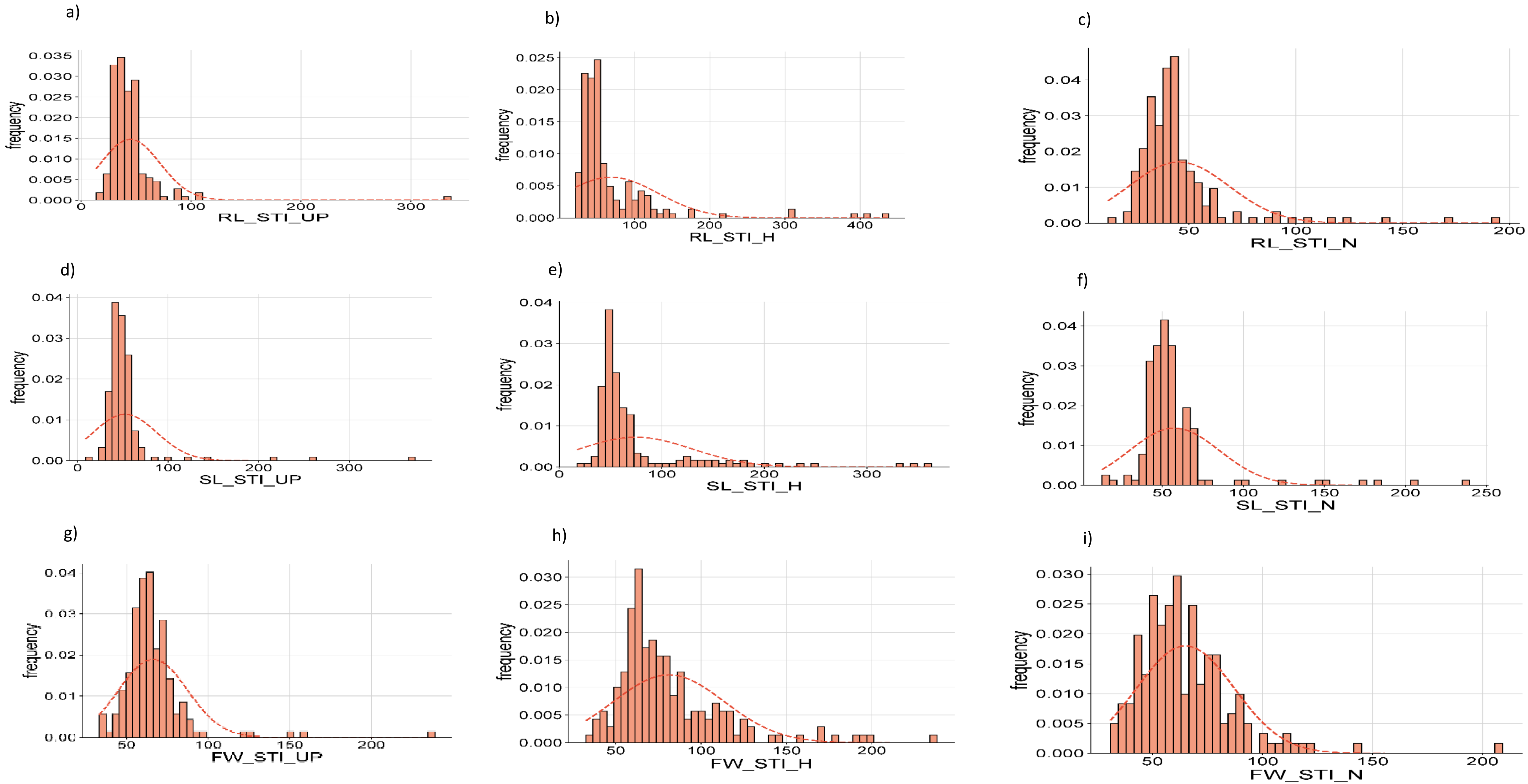

Figure S1: Frequency distribution of salt tolerance index (STI) of all the studied traits (root length (RL) , shoot length (SL) , fresh weight (FW), germination percentage(G%) , germination rate index (GRI), and germination pace(GP)) for all treatments (Unprimed (UP), Hydro Priming (H), and Nano Priming (N)) a) RL_STI_UP ,b) RL_STI_H, c) RL_STI_N, d) SL_STI_UP, e) SL_STI_H, f) SL_STI_N, g) FW_STI_UP, h) FW_STI_H, i) FW_STI_N, j) G%_STI_UP, k) G%_STI_H, l) G%_STI_N, m) GRI_STI_UP, n) GRI_STI_H , o) GRI_STI_N, p) GP_STI_UP, q) GP_STI_H, and r) GP_STI_N

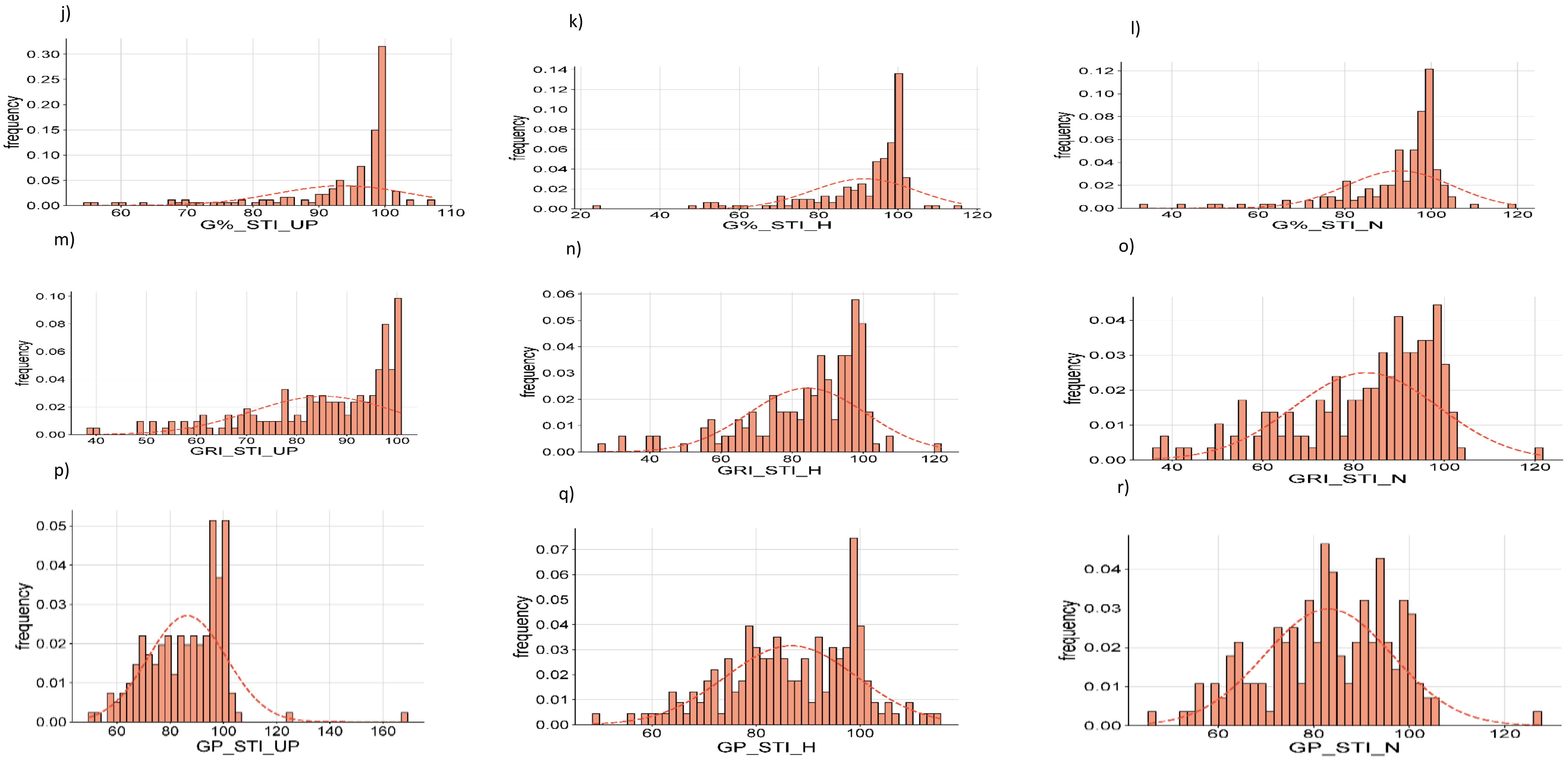

Figure S1 continued from the previous slide : Frequency distribution of salt tolerance index (STI) of all the studied traits (root length (RL) , shoot length (SL) , fresh weight (FW), germination percentage(G%) , germination rate index (GRI), and germination pace(GP)) for all treatments (Unprimed (UP), Hydro Priming (H), and Nano Priming (N)) a) RL_STI_UP ,b) RL_STI_H, c) RL_STI_N, d) SL_STI_UP, e) SL_STI_H, f) SL_STI_N, g) FW_STI_UP, h) FW_STI_H, i) FW_STI_N, j) G%_STI_UP, k) G%_STI_H, l) G%_STI_N, m) GRI_STI_UP, n) GRI_STI_H , o) GRI_STI_N, p) GP_STI_UP, q) GP_STI_H, and r) GP_STI_N

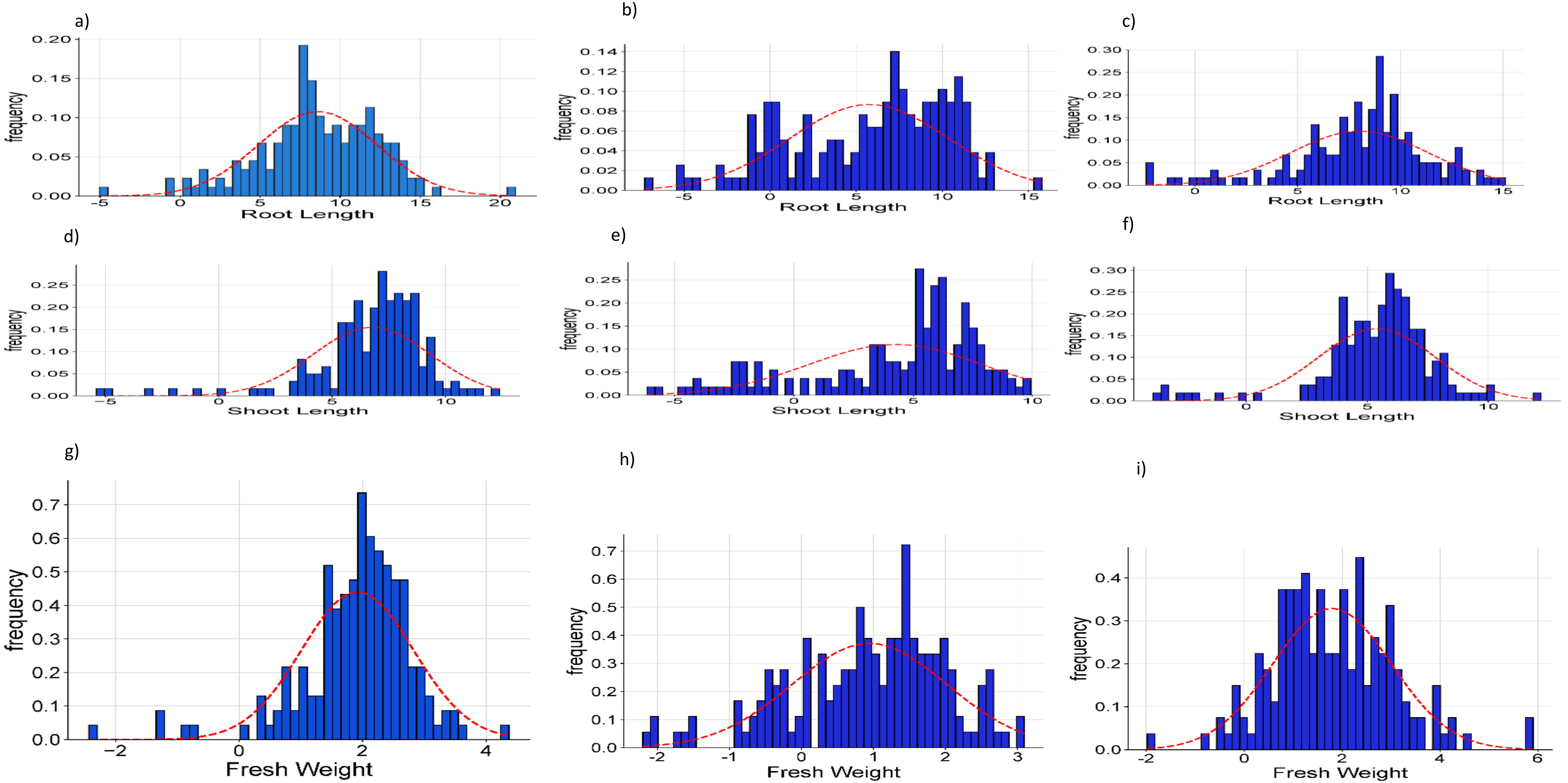

Figure S2: Frequency distribution of reduction indices (Red) for all the studied traits (root length (RL) , shoot length (SL) , fresh weight (FW), germination percentage(G%) , germination rate index (GRI), and germination pace(GP)) under control and salinity for all treatments (Unprimed (UP), Hydro Priming (H), and Nano Priming (N)) a) RL_Red_UP ,b) RL_Red_H, c) RL_Red_N, d) SL_Red_UP, e) SL_Red_H, f) SL_Red_N, g) FW_Red_UP, h) FW_Red_H, i) FW_Red_N, j) G%_Red_UP, k) G%_Red_H, l) G%_Red_N, m) GRI_Red_UP, n) GRI_Red_H , o) GRI_Red_N, p) GP_Red_UP, q) GP_Red_H, and r) GP_Red_N

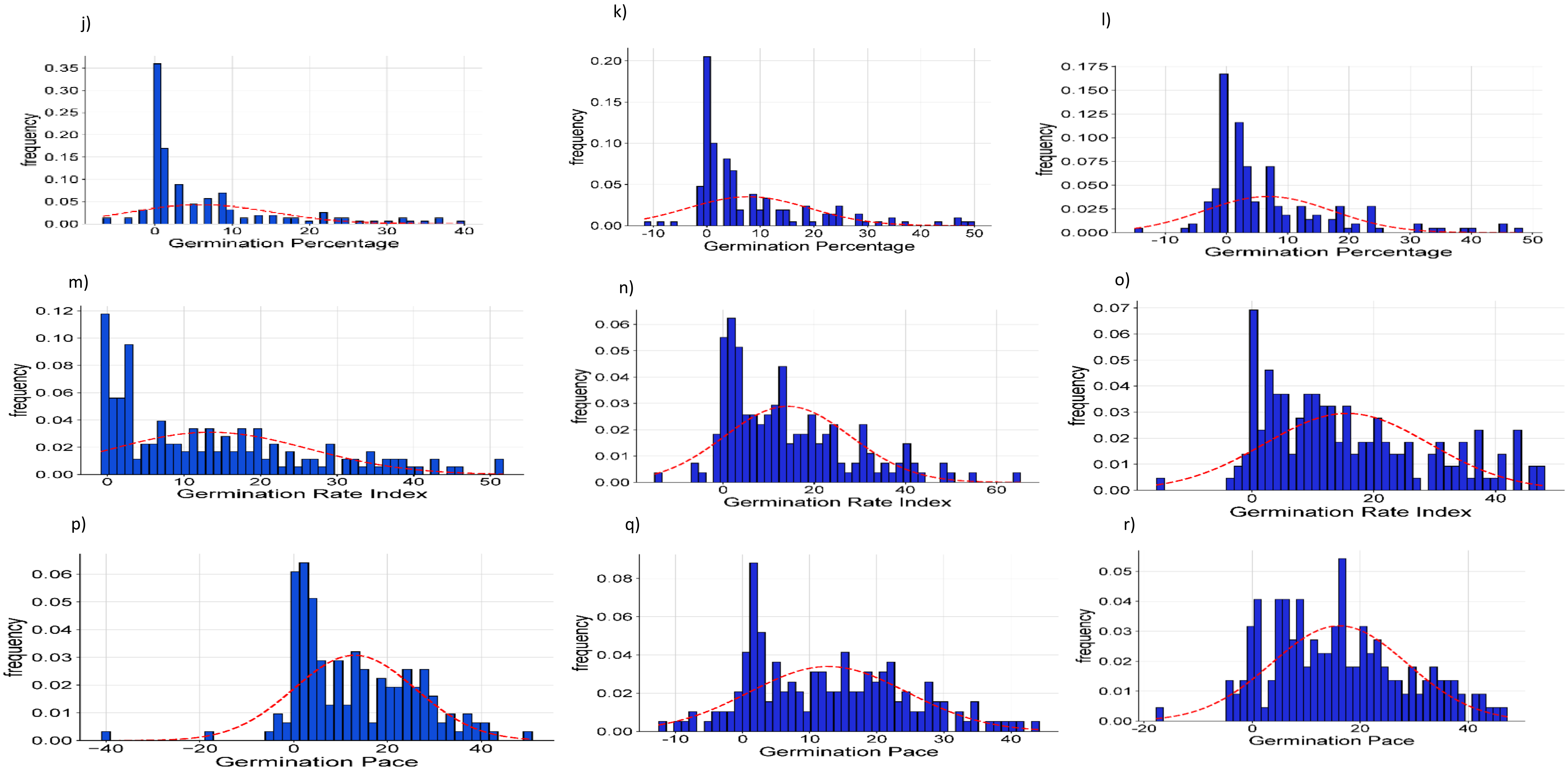

Figure S2 continued from the previous slide : Frequency distribution of reduction indices (Red) for all the studied traits (root length (RL) , shoot length (SL) , fresh weight (FW), germination percentage(G%) , germination rate index (GRI), and germination pace(GP)) under control and salinity for all treatments (Unprimed (UP), Hydro Priming (H), and Nano Priming (N)) a) RL_Red_UP ,b) RL_Red_H, c) RL_Red_N, d) SL_Red_UP, e) SL_Red_H, f) SL_Red_N, g) FW_Red_UP, h) FW_Red_H, i) FW_Red_N, j) G%_Red_UP, k) G%_Red_H, l) G%_Red_N, m) GRI_Red_UP, n) GRI_Red_H , o) GRI_Red_N, p) GP_Red_UP, q) GP_Red_H, and r) GP_Red_N

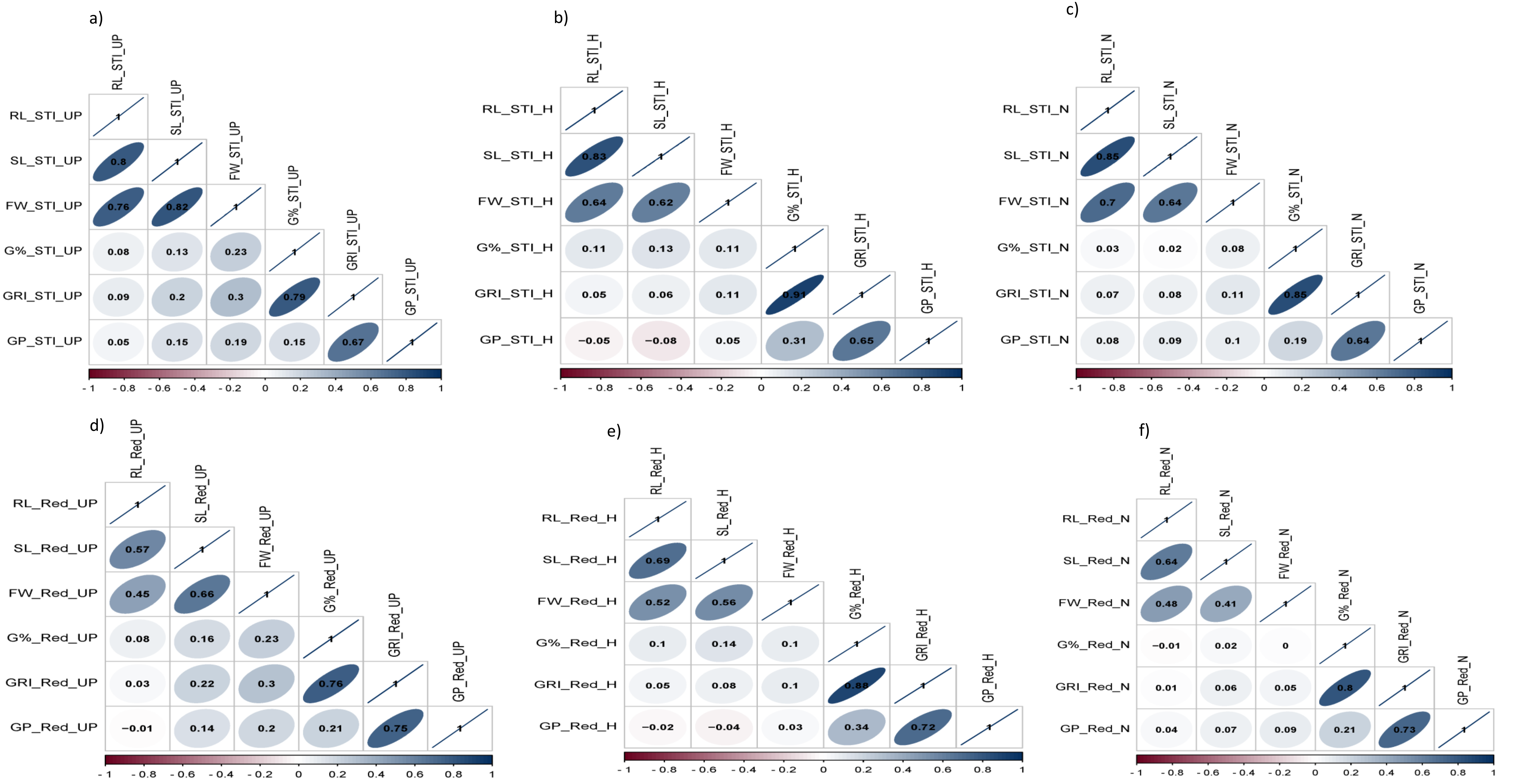

Figure S3: Correlation analysis of salt tolerance index (STI) and reduction (Red) for all the studied traits (root length (RL) , shoot length (SL) , fresh weight (FW), germination percentage(G%) , germination rate index (GRI), and germination pace(GP) for all treatments (Unprimed (UP), Hydro Priming (H), and Nano Priming (N)) : for salt tolerance index a) unprimed , b) hydro priming, and c) nano priming; for reduction d) unprimed , e) hydro priming, and f) nano priming

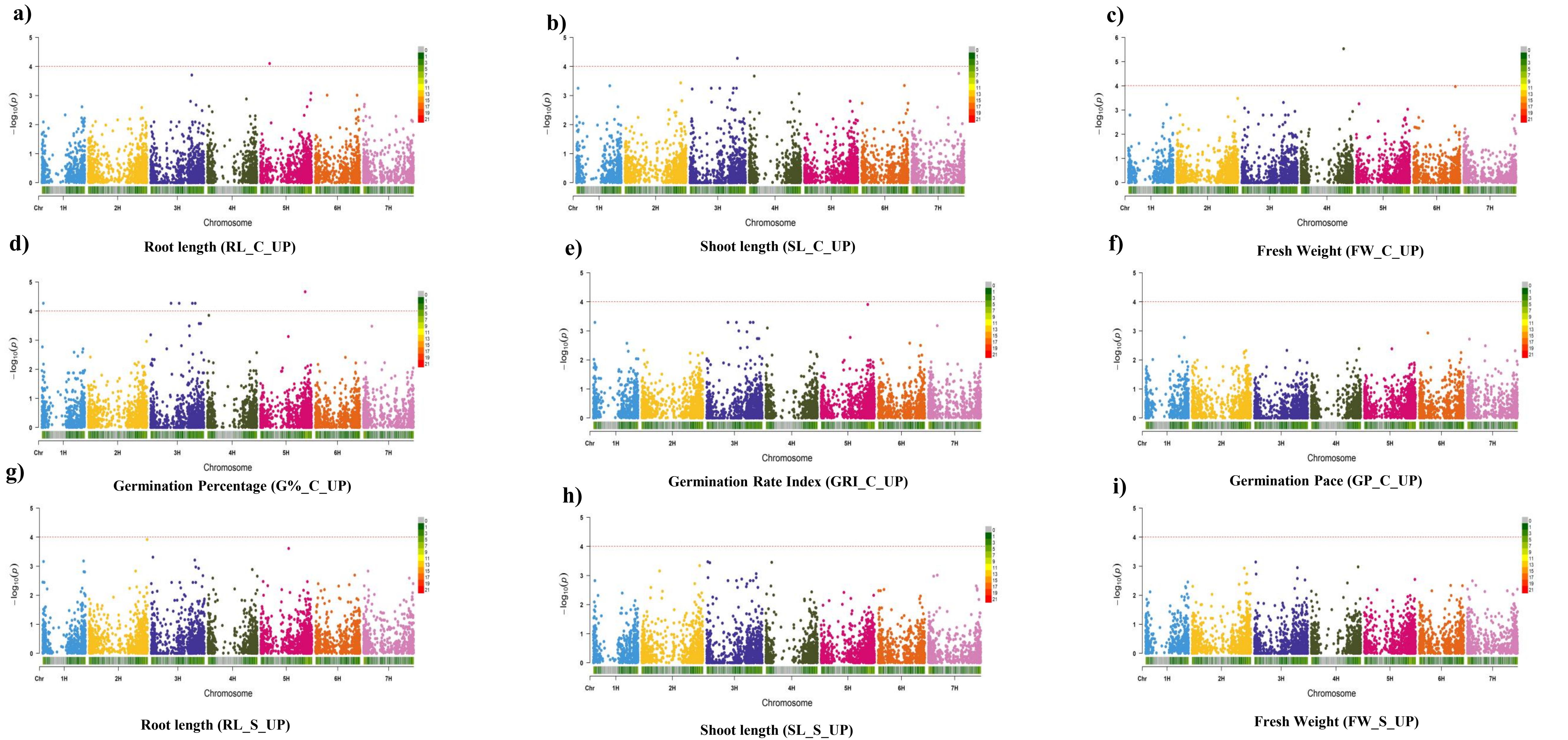

Figure S4: Manhattan plot under control (C), salinity (S), salt tolerance index (STI), and reduction (Red) for unprimed conditions (UP): under control a)Root Length, b)Shoot Length, c)Fresh Weight, d)Germination Percentage, e)Germination Rate Index, and f)Germination Pace ; under salinity g)Root Length, h)Shoot Length, i)Fresh Weight, j)Germination Percentage, k)Germination Rate Index, and l)Germination Pace; for salt tolerance index m)Root Length, n)Shoot Length, o)Fresh Weight, p)Germination Percentage, q)Germination Rate Index, and r)Germination Pace; for reduction s)Root Length, t)Shoot Length, u)Fresh Weight, v)Germination Percentage, w)Germination Rate Index, and x)Germination Pace. Each color indicates a different chromosome, the x-axis shows the chromosome number, the y-axis shows the -log10 (p), and the dots above the red line are significant markers at –log10 (P) ≥ 4.0.

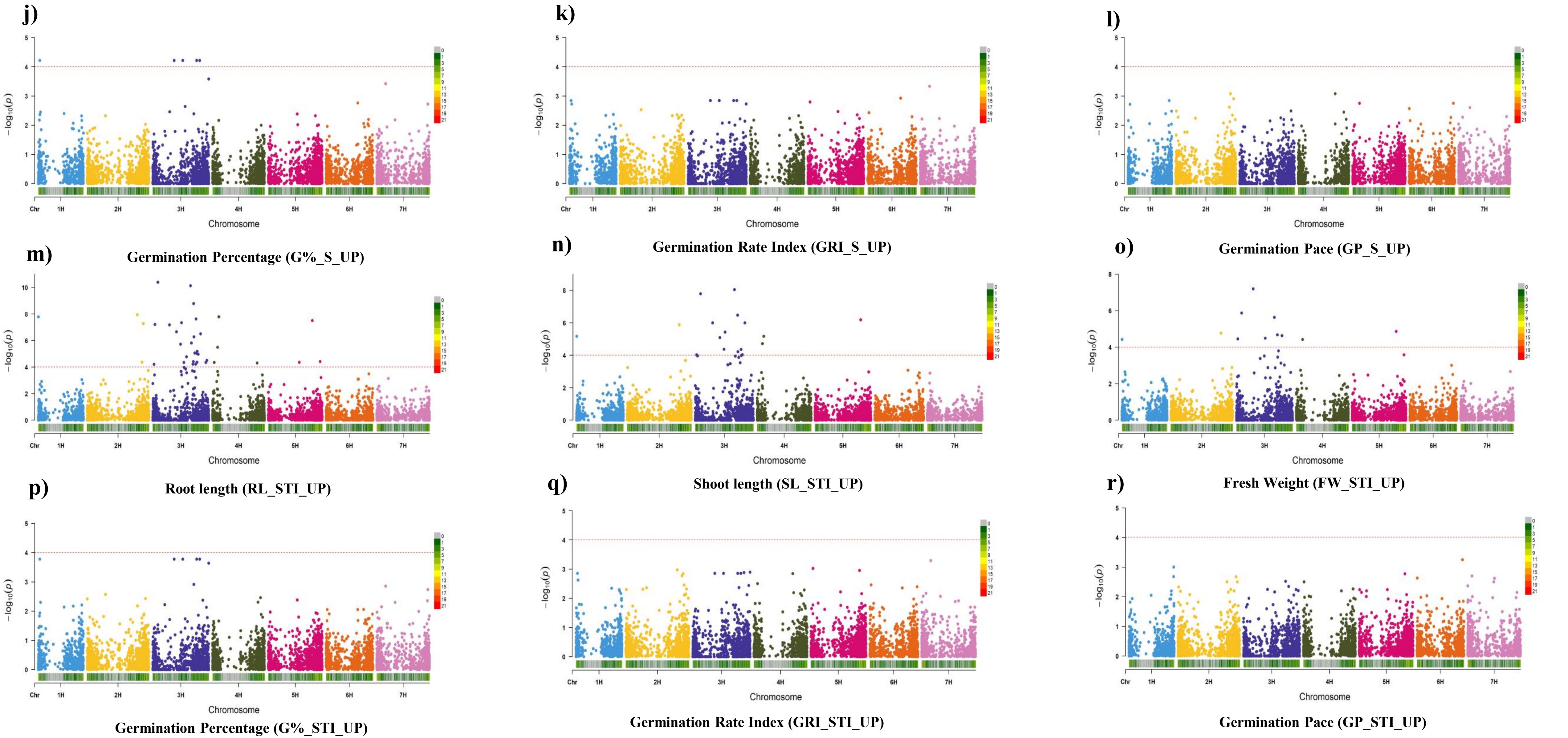

Figure S4 continued from the previous slide: Manhattan plot under control (C), salinity (S), salt tolerance index (STI), and reduction (Red) for unprimed conditions (UP): under control a)Root Length, b)Shoot Length, c)Fresh Weight, d)Germination Percentage, e)Germination Rate Index, and f)Germination Pace ; under salinity g)Root Length, h)Shoot Length, i)Fresh Weight, j)Germination Percentage, k)Germination Rate Index, and l)Germination Pace; for salt tolerance index m)Root Length, n)Shoot Length, o)Fresh Weight, p)Germination Percentage, q)Germination Rate Index, and r)Germination Pace; for reduction s)Root Length, t)Shoot Length, u)Fresh Weight, v)Germination Percentage, w)Germination Rate Index, and x)Germination Pace. Each color indicates a different chromosome, the x-axis shows the chromosome number, the y-axis shows the -log10 (p), and the dots above the red line are significant markers at –log10 (P) ≥ 4.0.

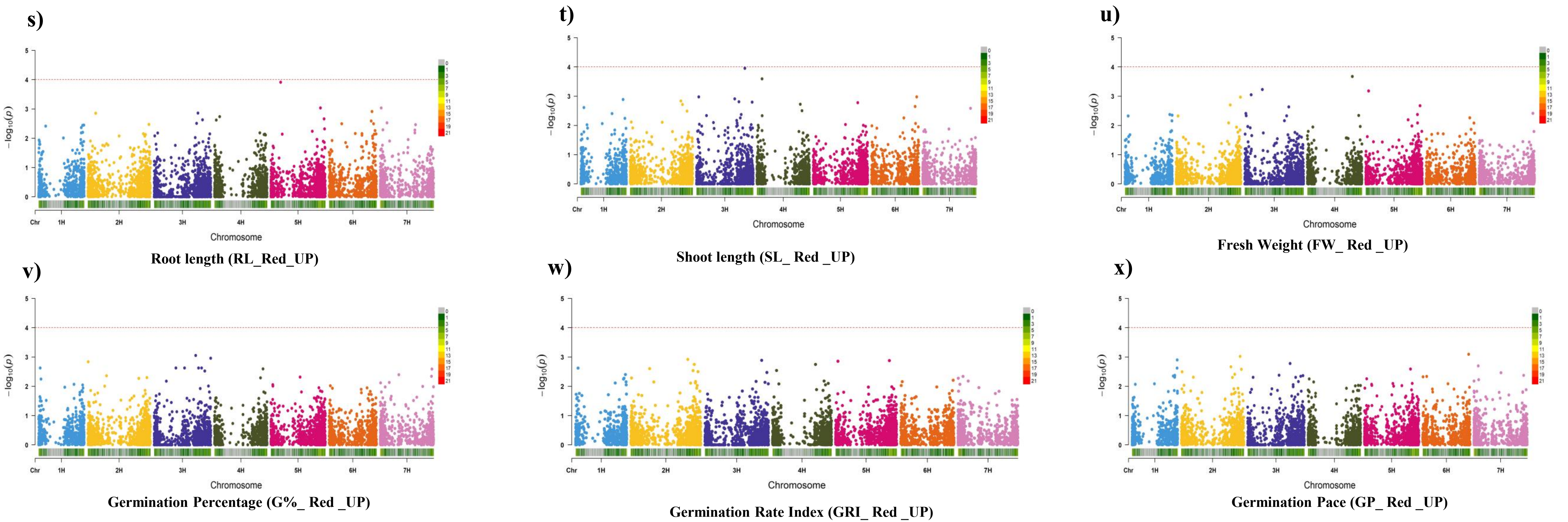

Figure S4 continued from the previous slide: Manhattan plot under control (C), salinity (S), salt tolerance index (STI), and reduction (Red) for unprimed conditions (UP): under control a)Root Length, b)Shoot Length, c)Fresh Weight, d)Germination Percentage, e)Germination Rate Index, and f)Germination Pace ; under salinity g)Root Length, h)Shoot Length, i)Fresh Weight, j)Germination Percentage, k)Germination Rate Index, and l)Germination Pace; for salt tolerance index m)Root Length, n)Shoot Length, o)Fresh Weight, p)Germination Percentage, q)Germination Rate Index, and r)Germination Pace; for reduction s)Root Length, t)Shoot Length, u)Fresh Weight, v)Germination Percentage, w)Germination Rate Index, and x)Germination Pace. Each color indicates a different chromosome, the x-axis shows the chromosome number, the y-axis shows the –log10 (p), and the dots above the red line are significant markers at –log10 (P) ≥ 4.0

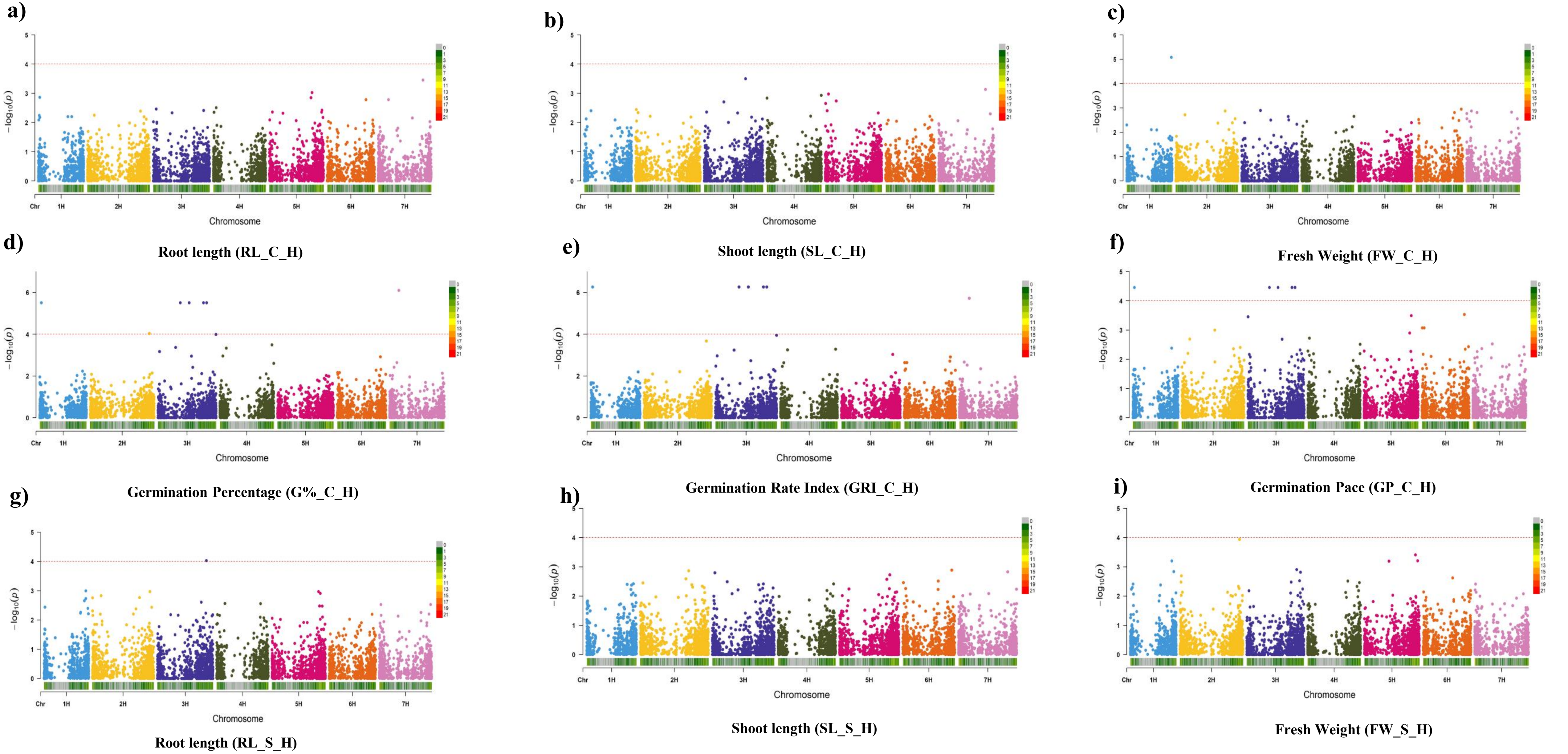

Figure S5: Manhattan plot under control (C), salinity (S), salt tolerance index (STI), and reduction (Red) for hydropriming conditions (UP): under control a)Root Length, b)Shoot Length, c)Fresh Weight, d)Germination Percentage, e)Germination Rate Index, and f)Germination Pace ; under salinity g)Root Length, h)Shoot Length, i)Fresh Weight, j)Germination Percentage, k)Germination Rate Index, and l)Germination Pace; for salt tolerance index m)Root Length, n)Shoot Length, o)Fresh Weight, p)Germination Percentage, q)Germination Rate Index, and r)Germination Pace; for reduction s)Root Length, t)Shoot Length, u)Fresh Weight, v)Germination Percentage, w)Germination Rate Index, and x)Germination Pace. Each color indicates a different chromosome, the x-axis shows the chromosome number, the y-axis shows the -log10 (p), and the dots above the red line are significant markers at –log10 (P) ≥ 4.0.

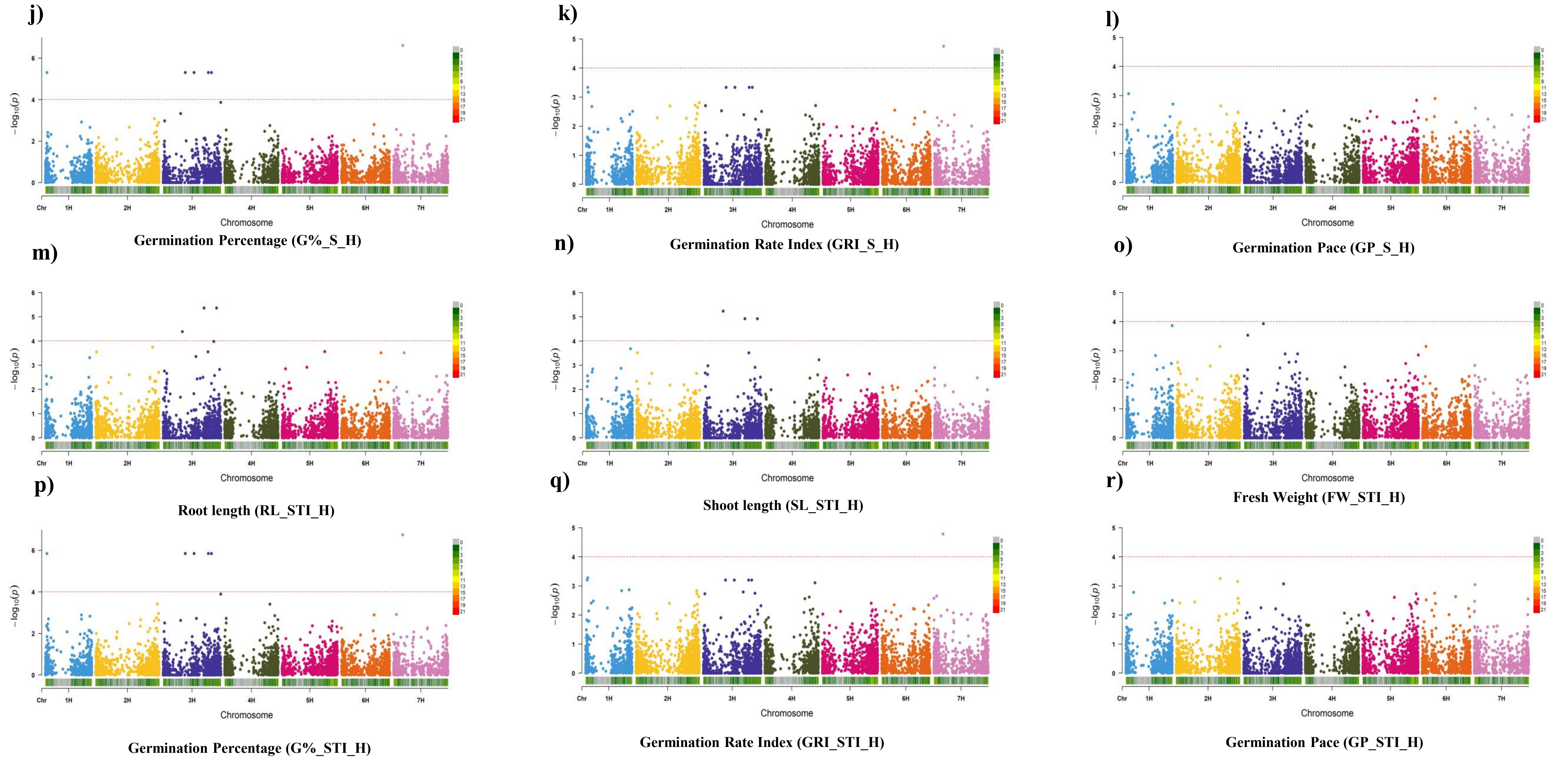

Figure S5 continued from the previous slide : Manhattan plot under control (C), salinity (S), salt tolerance index (STI), and reduction (Red) for hydropriming conditions (UP): under control a)Root Length, b)Shoot Length, c)Fresh Weight, d)Germination Percentage, e)Germination Rate Index, and f)Germination Pace ; under salinity g)Root Length, h)Shoot Length, i)Fresh Weight, j)Germination Percentage, k)Germination Rate Index, and l)Germination Pace; for salt tolerance index m)Root Length, n)Shoot Length, o)Fresh Weight, p)Germination Percentage, q)Germination Rate Index, and r)Germination Pace; for reduction s)Root Length, t)Shoot Length, u)Fresh Weight, v)Germination Percentage, w)Germination Rate Index, and x)Germination Pace. Each color indicates a different chromosome, the x-axis shows the chromosome number, the y-axis shows the -log10 (p), and the dots above the red line are significant markers at –log10 (P) ≥ 4.0

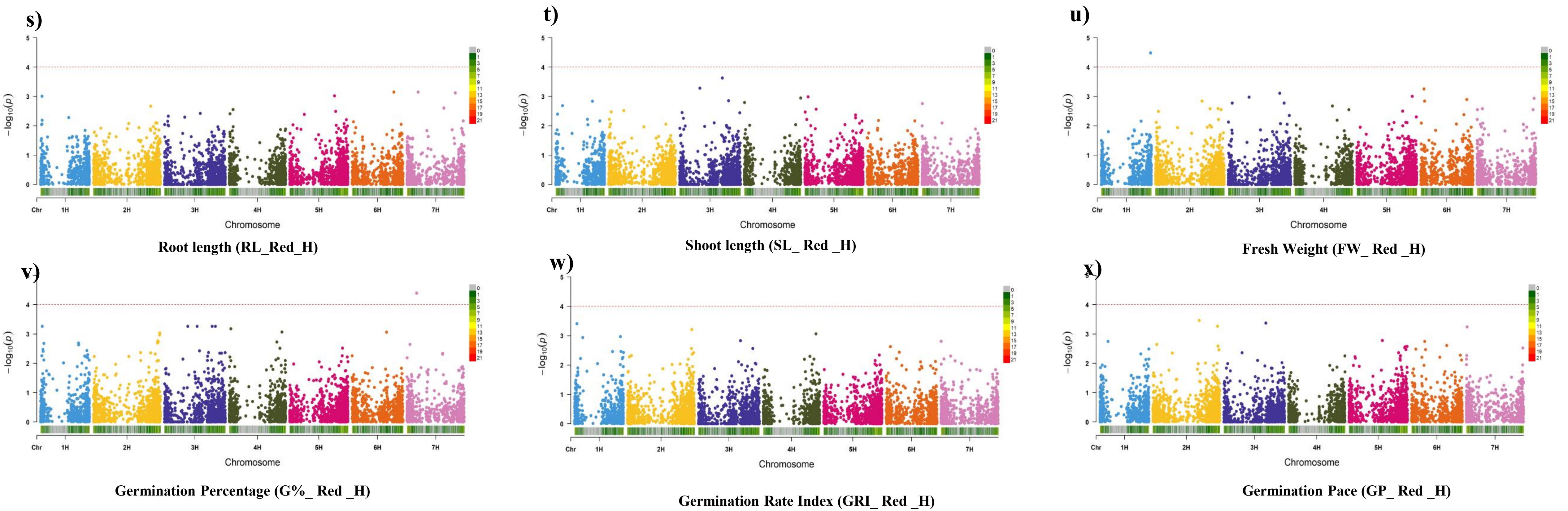

Figure S5 continued from the previous slide : Manhattan plot under control (C), salinity (S), salt tolerance index (STI), and reduction (Red) for hydropriming conditions (UP): under control a)Root Length, b)Shoot Length, c)Fresh Weight, d)Germination Percentage, e)Germination Rate Index, and f)Germination Pace ; under salinity g)Root Length, h)Shoot Length, i)Fresh Weight, j)Germination Percentage, k)Germination Rate Index, and l)Germination Pace; for salt tolerance index m)Root Length, n)Shoot Length, o)Fresh Weight, p)Germination Percentage, q)Germination Rate Index, and r)Germination Pace; for reduction s)Root Length, t)Shoot Length, u)Fresh Weight, v)Germination Percentage, w)Germination Rate Index, and x)Germination Pace. Each color indicates a different chromosome, the x-axis shows the chromosome number, the y-axis shows the -log10 (p), and the dots above the red line are significant markers at –log10 (P) ≥ 4.0

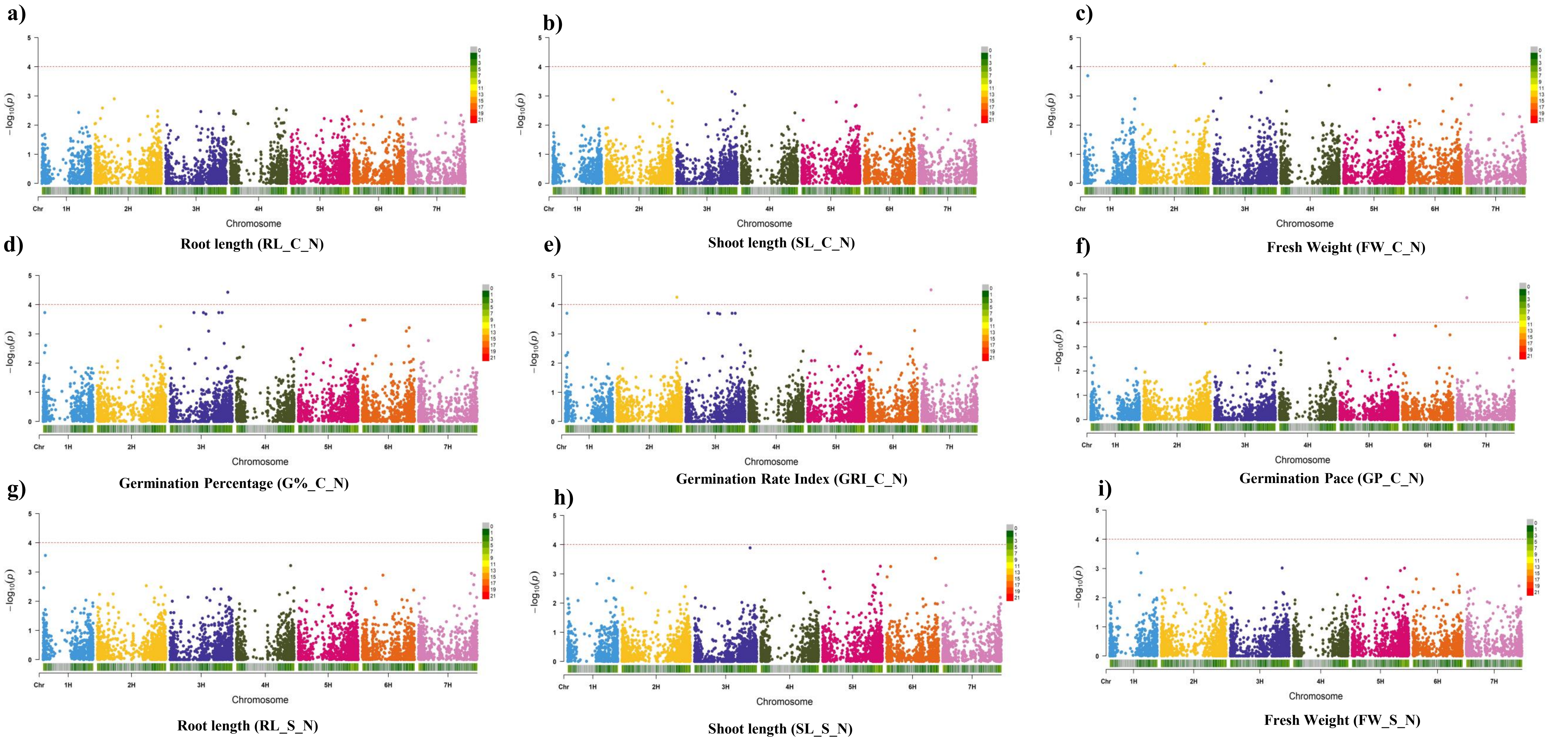

Figure S6: Manhattan plot under control (C), salinity (S), salt tolerance index (STI), and reduction (Red) for nano priming conditions (UP): under control a)Root Length, b)Shoot Length, c)Fresh Weight, d)Germination Percentage, e)Germination Rate Index, and f)Germination Pace ; under salinity g)Root Length, h)Shoot Length, i)Fresh Weight, j)Germination Percentage, k)Germination Rate Index, and l)Germination Pace; for salt tolerance index m)Root Length, n)Shoot Length, o)Fresh Weight, p)Germination Percentage, q)Germination Rate Index, and r)Germination Pace; for reduction s)Root Length, t)Shoot Length, u)Fresh Weight, v)Germination Percentage, w)Germination Rate Index, and x)Germination Pace. Each color indicates a different chromosome, the x-axis shows the chromosome number, the y-axis shows the -log10 (p), and the dots above the red line are significant markers at –log10 (P) ≥ 4.0.

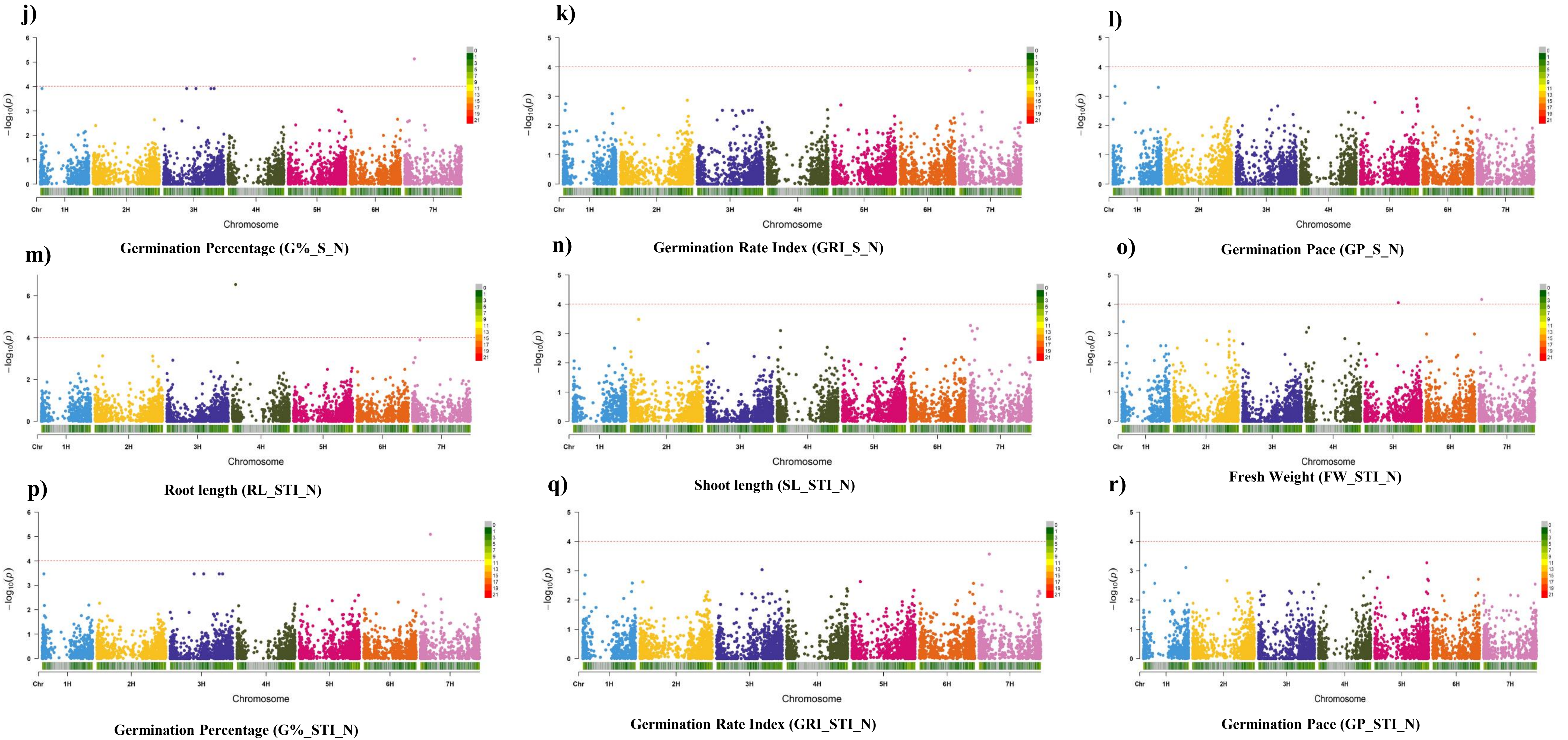

Figure S6 continued from the previous slide : Manhattan plot under control (C), salinity (S), salt tolerance index (STI), and reduction (Red) for nano priming conditions (UP): under control a)Root Length, b)Shoot Length, c)Fresh Weight, d)Germination Percentage, e)Germination Rate Index, and f)Germination Pace ; under salinity g)Root Length, h)Shoot Length, i)Fresh Weight, j)Germination Percentage, k)Germination Rate Index, and l)Germination Pace; for salt tolerance index m)Root Length, n)Shoot Length, o)Fresh Weight, p)Germination Percentage, q)Germination Rate Index, and r)Germination Pace; for reduction s)Root Length, t)Shoot Length, u)Fresh Weight, v)Germination Percentage, w)Germination Rate Index, and x)Germination Pace. Each color indicates a different chromosome, the x-axis shows the chromosome number, the y-axis shows the -log10 (p), and the dots above the red line are significant markers at –log10 (P) ≥ 4.0

s)

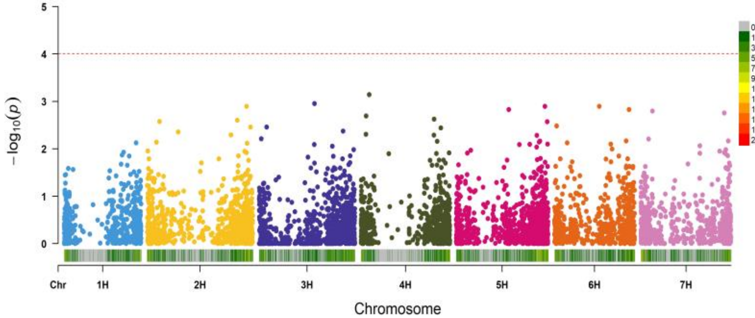

Root length (RL_Red_N)

t)

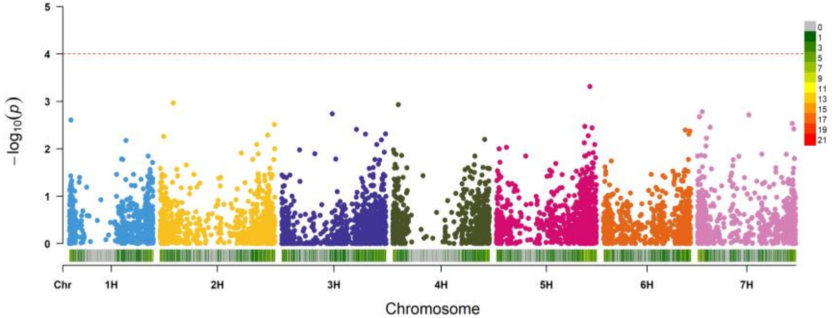

Shoot length (SL_ Red _N)

u)

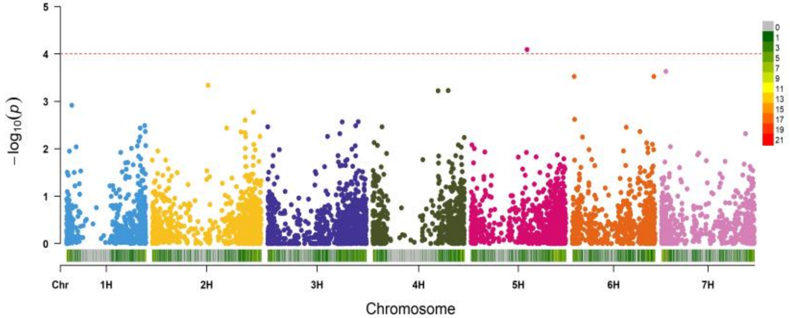

Fresh Weight (FW_ Red _N)

v)

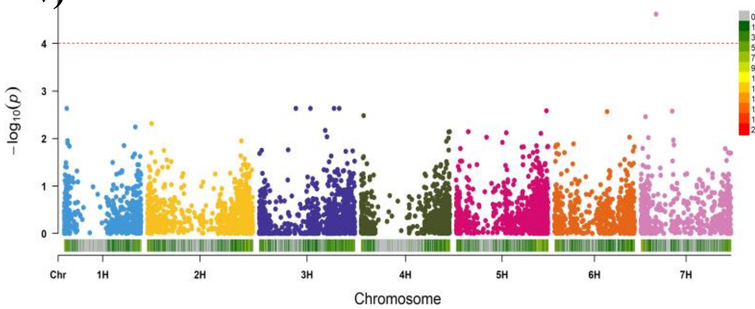

Germination Percentage (G%_ Red _N)

w)

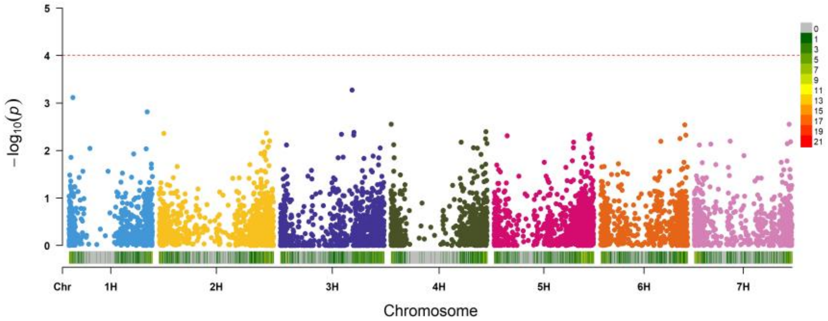

Germination Rate Index (GRI_ Red _N)

x)

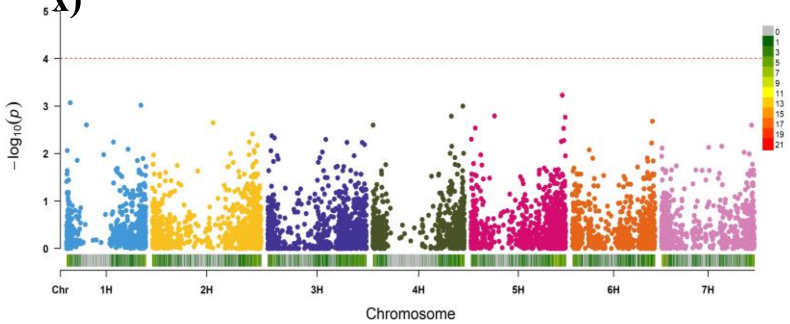

Germination Pace (GP_ Red _N)

Figure S6 continued from the previous slide : Manhattan plot under control (C), salinity (S), salt tolerance index (STI), and reduction (Red) for nano priming conditions (UP): under control a)Root Length, b)Shoot Length, c)Fresh Weight, d)Germination Percentage, e)Germination Rate Index, and f)Germination Pace ; under salinity g)Root Length, h)Shoot Length, i)Fresh Weight, j)Germination Percentage, k)Germination Rate Index, and l)Germination Pace; for salt tolerance index m)Root Length, n)Shoot Length, o)Fresh Weight, p)Germination Percentage, q)Germination Rate Index, and r)Germination Pace; for reduction s)Root Length, t)Shoot Length, u)Fresh Weight, v)Germination Percentage, w)Germination Rate Index, and x)Germination Pace. Each color indicates a different chromosome, the x-axis shows the chromosome number, the y-axis shows the -log10 (p), and the dots above the red line are significant markers at –log10 (P) ≥ 4.0

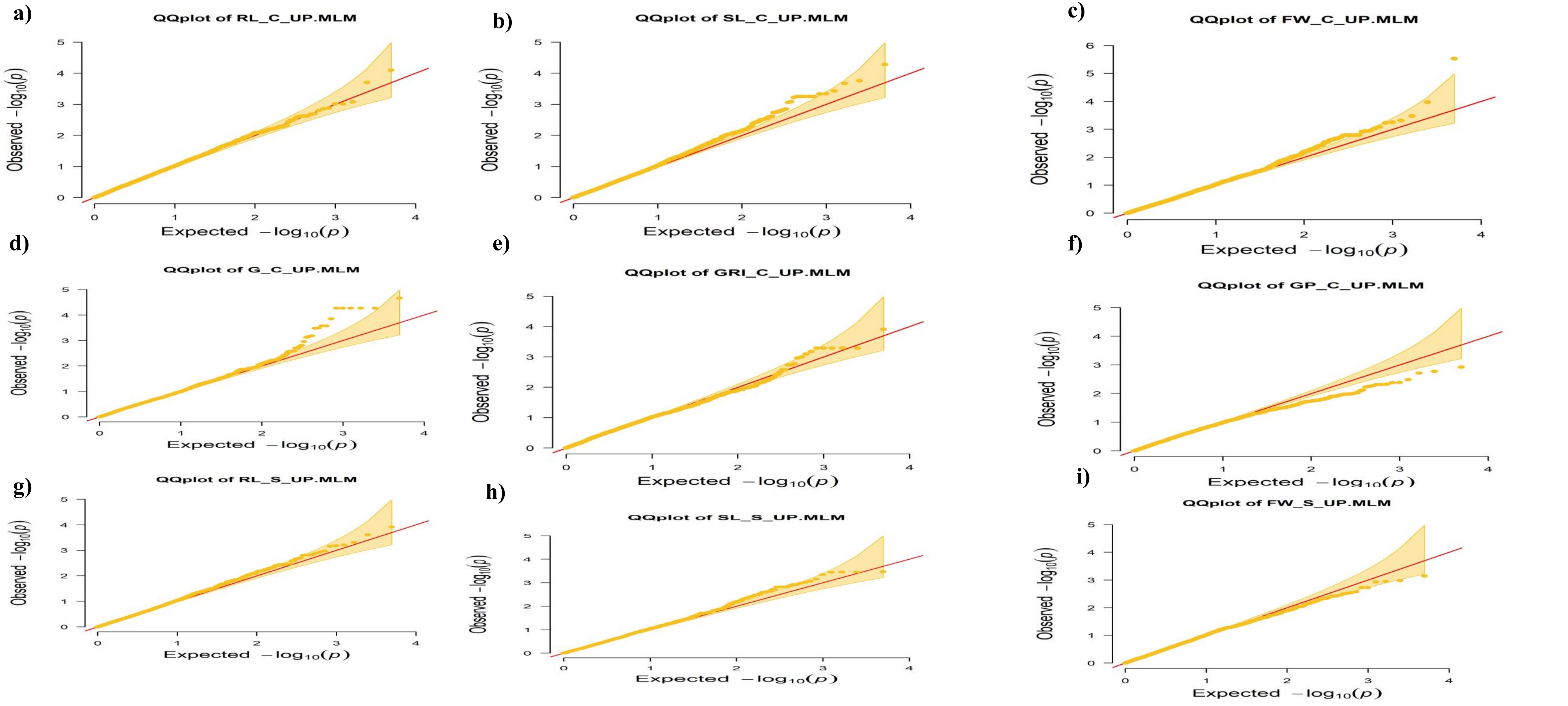

Figure S7: Quantile Quantile plot under control (C), salinity (S), salt tolerance index (STI), and reduction (Red) for unprimed conditions (UP): under control a)Root Length, b)Shoot Length, c)Fresh Weight, d)Germination Percentage, e)Germination Rate Index, and f)Germination Pace ; under salinity g)Root Length, h)Shoot Length, i)Fresh Weight, j)Germination Percentage, k)Germination Rate Index, and l)Germination Pace; for salt tolerance index m)Root Length, n)Shoot Length, o)Fresh Weight, p)Germination Percentage, q)Germination Rate Index, and r)Germination Pace; for reduction s)Root Length, t)Shoot Length, u)Fresh Weight, v)Germination Percentage, w)Germination Rate Index, and x)Germination Pace. The x-axis shows the expected –log10 (P), the y-axis shows the observed -log10 (p).

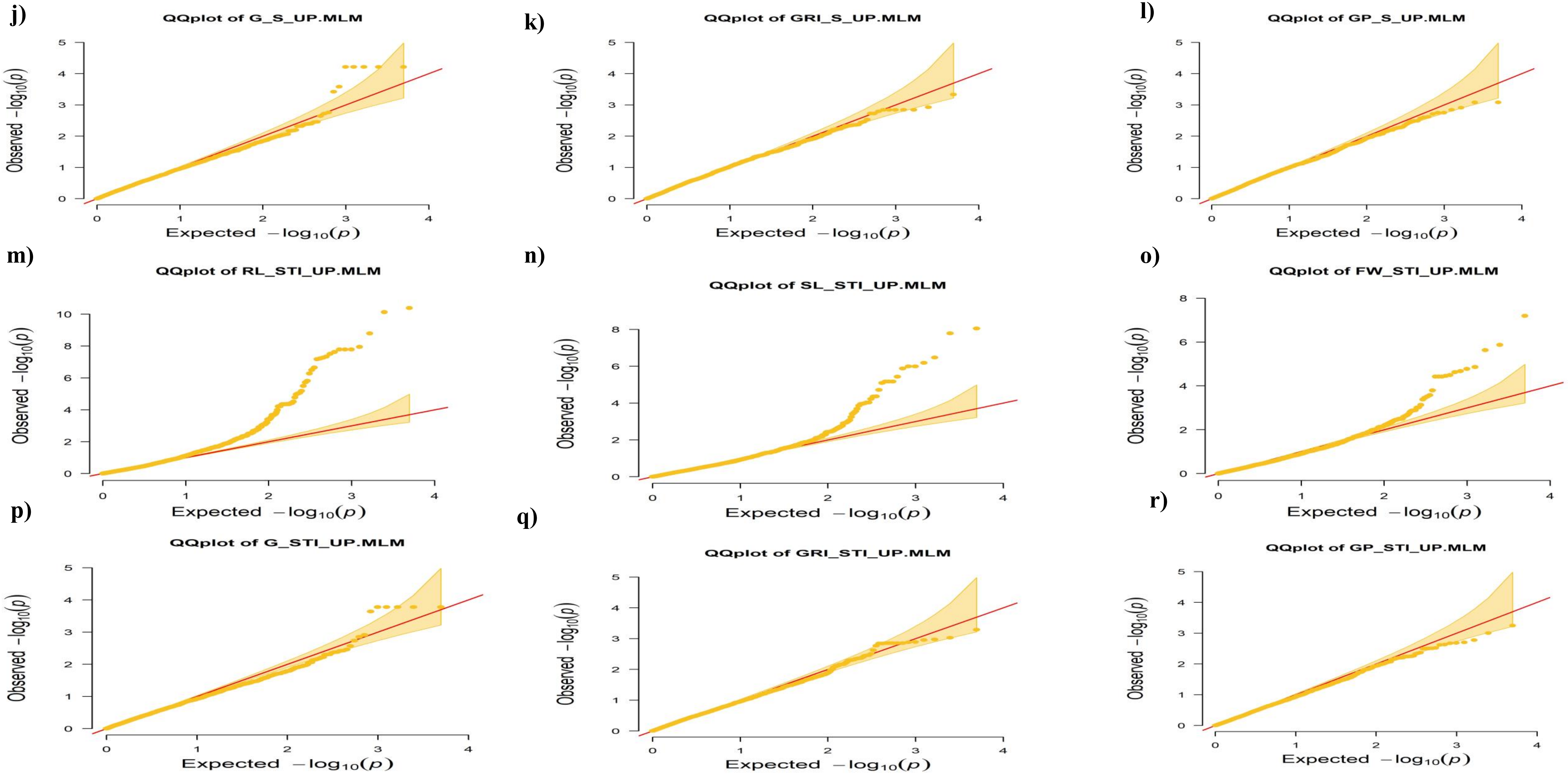

Figure S7 continued from the previous slide : Quantile Quantile plot under control (C), salinity (S), salt tolerance index (STI), and reduction (Red) for unprimed conditions (UP): under control a)Root Length, b)Shoot Length, c)Fresh Weight, d)Germination Percentage, e)Germination Rate Index, and f)Germination Pace ; under salinity g)Root Length, h)Shoot Length, i)Fresh Weight, j)Germination Percentage, k)Germination Rate Index, and l)Germination Pace; for salt tolerance index m)Root Length, n)Shoot Length, o)Fresh Weight, p)Germination Percentage, q)Germination Rate Index, and r)Germination Pace; for reduction s)Root Length, t)Shoot Length, u)Fresh Weight, v)Germination Percentage, w)Germination Rate Index, and x)Germination Pace. The x-axis shows the expected –log10 (P), the y-axis shows the observed -log10 (p).

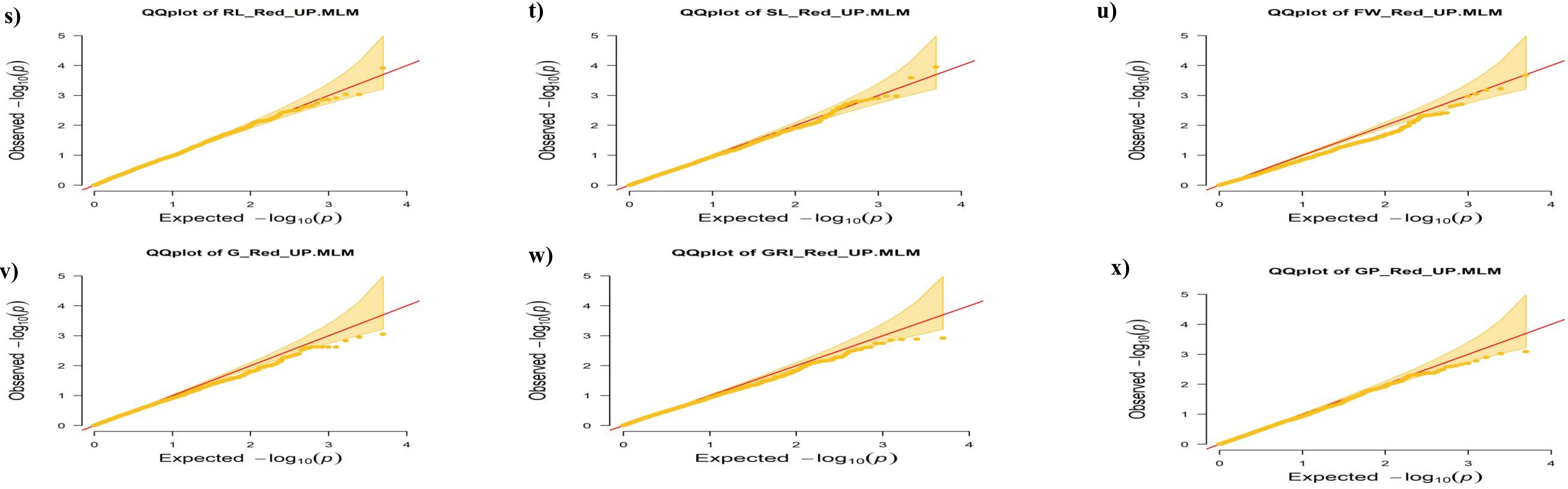

Figure S7 continued from the previous slide : Quantile Quantile plot under control (C), salinity (S), salt tolerance index (STI), and reduction (Red) for unprimed conditions (UP): under control a)Root Length, b)Shoot Length, c)Fresh Weight, d)Germination Percentage, e)Germination Rate Index, and f)Germination Pace ; under salinity g)Root Length, h)Shoot Length, i)Fresh Weight, j)Germination Percentage, k)Germination Rate Index, and l)Germination Pace; for salt tolerance index m)Root Length, n)Shoot Length, o)Fresh Weight, p)Germination Percentage, q)Germination Rate Index, and r)Germination Pace; for reduction s)Root Length, t)Shoot Length, u)Fresh Weight, v)Germination Percentage, w)Germination Rate Index, and x)Germination Pace. The x-axis shows the expected –log10 (P), the y-axis shows the observed -log10 (p).

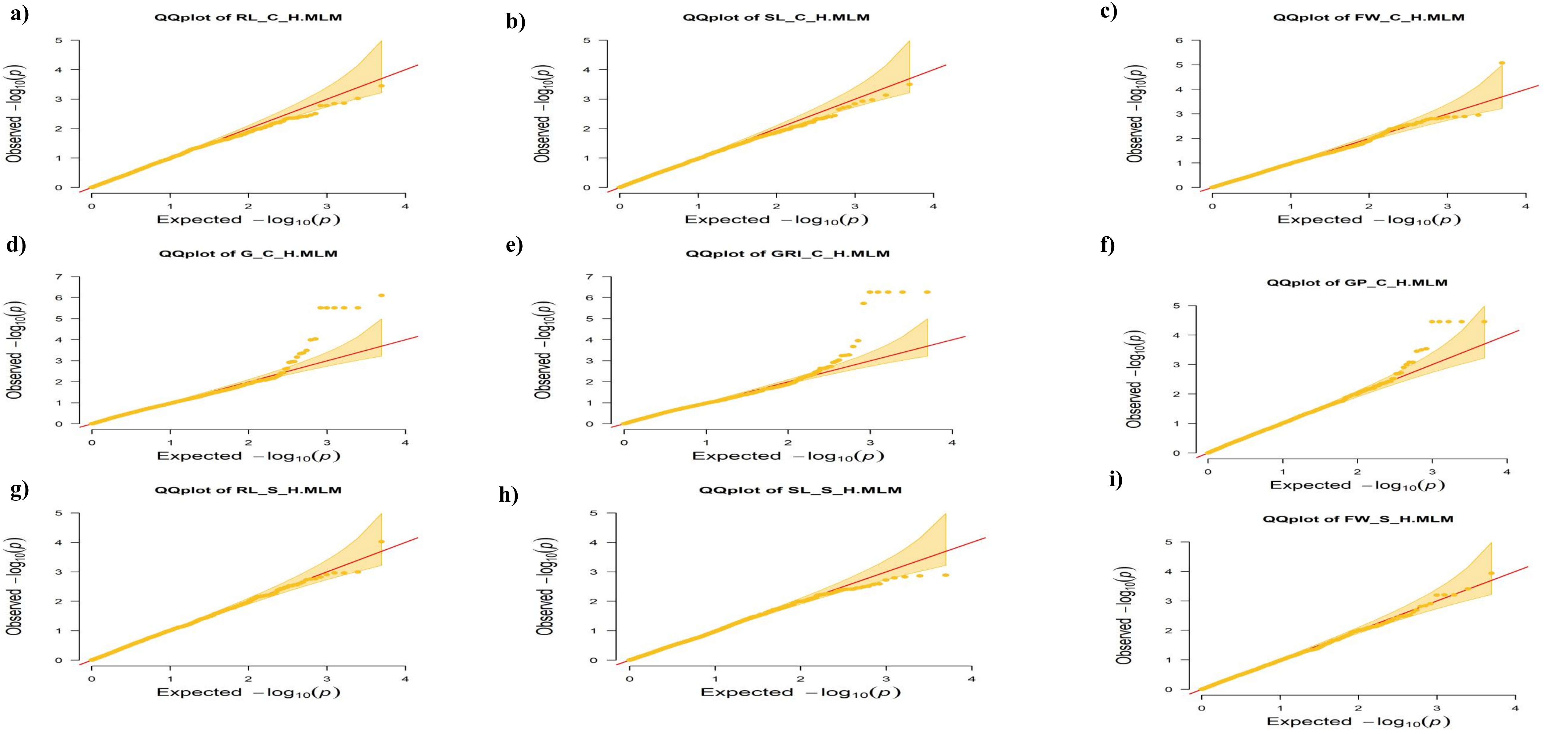

Figure S8: Quantile Quantile plot under control (C), salinity (S), salt tolerance index (STI), and reduction (Red) for hydropriming conditions (UP): under control a)Root Length, b)Shoot Length, c)Fresh Weight, d)Germination Percentage, e)Germination Rate Index, and f)Germination Pace ; under salinity g)Root Length, h)Shoot Length, i)Fresh Weight, j)Germination Percentage, k)Germination Rate Index, and l)Germination Pace; for salt tolerance index m)Root Length, n)Shoot Length, o)Fresh Weight, p)Germination Percentage, q)Germination Rate Index, and r)Germination Pace; for reduction s)Root Length, t)Shoot Length, u)Fresh Weight, v)Germination Percentage, w)Germination Rate Index, and x)Germination Pace. The x-axis shows the expected –log10 (P), the y-axis shows the observed -log10 (p).

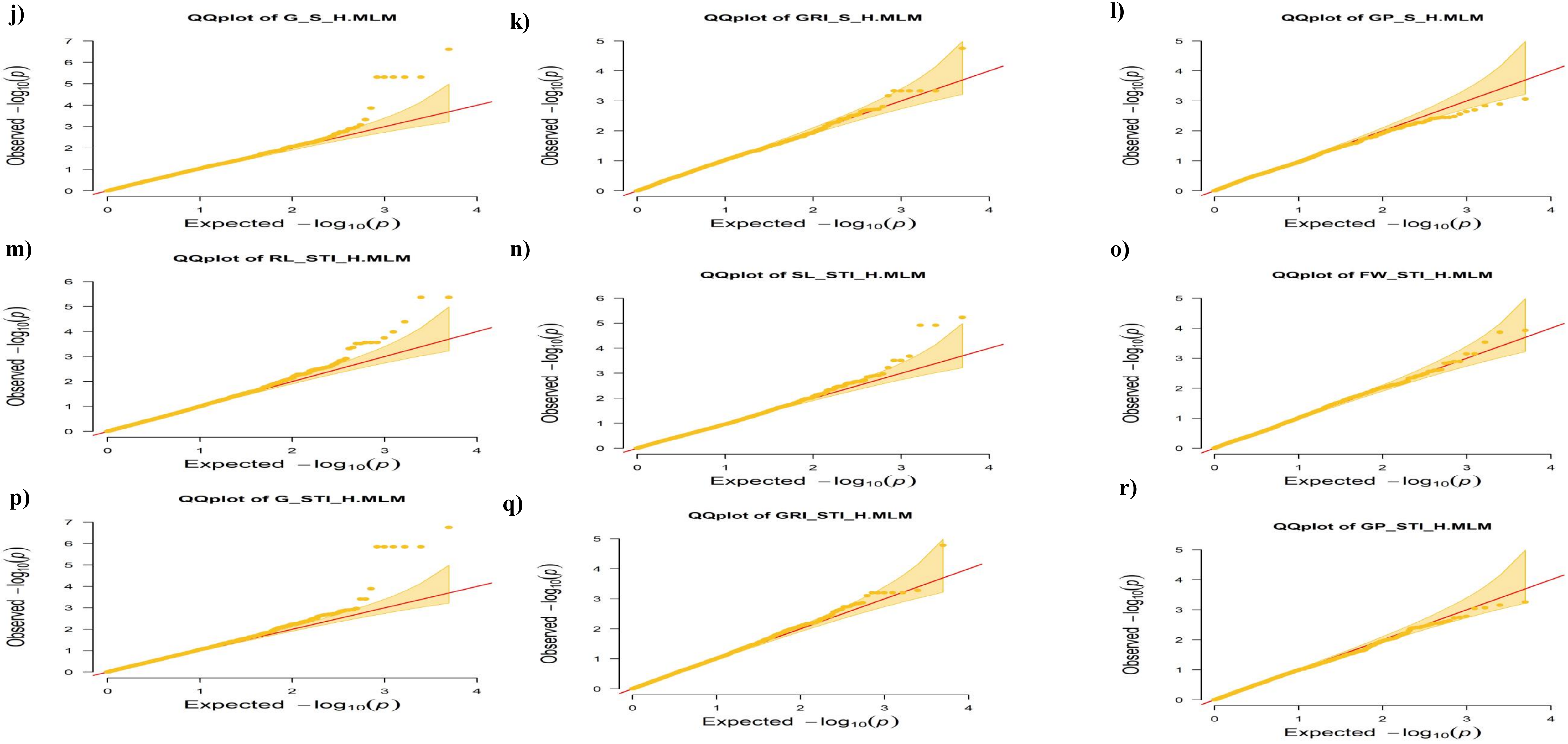

Figure S8 continued from the previous slide : Quantile Quantile plot under control (C), salinity (S), salt tolerance index (STI), and reduction (Red) for hydropriming conditions (UP): under control a)Root Length, b)Shoot Length, c)Fresh Weight, d)Germination Percentage, e)Germination Rate Index, and f)Germination Pace ; under salinity g)Root Length, h)Shoot Length, i)Fresh Weight, j)Germination Percentage, k)Germination Rate Index, and l)Germination Pace; for salt tolerance index m)Root Length, n)Shoot Length, o)Fresh Weight, p)Germination Percentage, q)Germination Rate Index, and r)Germination Pace; for reduction s)Root Length, t)Shoot Length, u)Fresh Weight, v)Germination Percentage, w)Germination Rate Index, and x)Germination Pace. The x-axis shows the expected –log10 (P), the y-axis shows the observed -log10 (p)

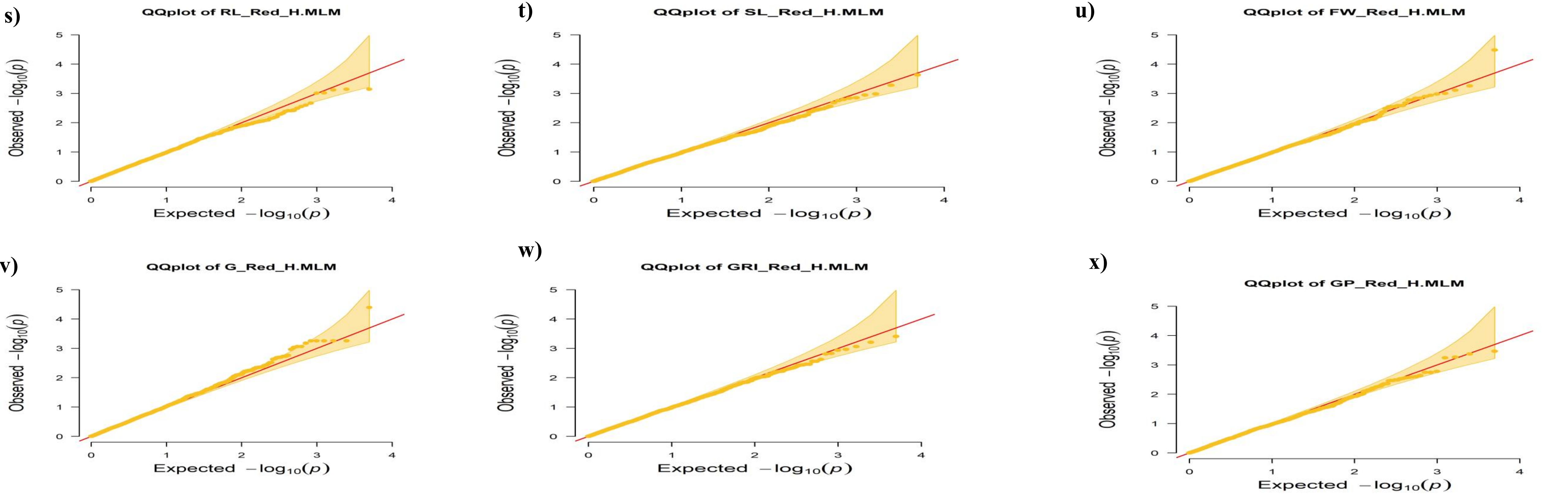

Figure S8 continued from the previous slide : Quantile Quantile plot under control (C), salinity (S), salt tolerance index (STI), and reduction (Red) for hydropriming conditions (UP): under control a)Root Length, b)Shoot Length, c)Fresh Weight, d)Germination Percentage, e)Germination Rate Index, and f)Germination Pace ; under salinity g)Root Length, h)Shoot Length, i)Fresh Weight, j)Germination Percentage, k)Germination Rate Index, and l)Germination Pace; for salt tolerance index m)Root Length, n)Shoot Length, o)Fresh Weight, p)Germination Percentage, q)Germination Rate Index, and r)Germination Pace; for reduction s)Root Length, t)Shoot Length, u)Fresh Weight, v)Germination Percentage, w)Germination Rate Index, and x)Germination Pace. The x-axis shows the expected –log10 (P), the y-axis shows the observed -log10 (p)

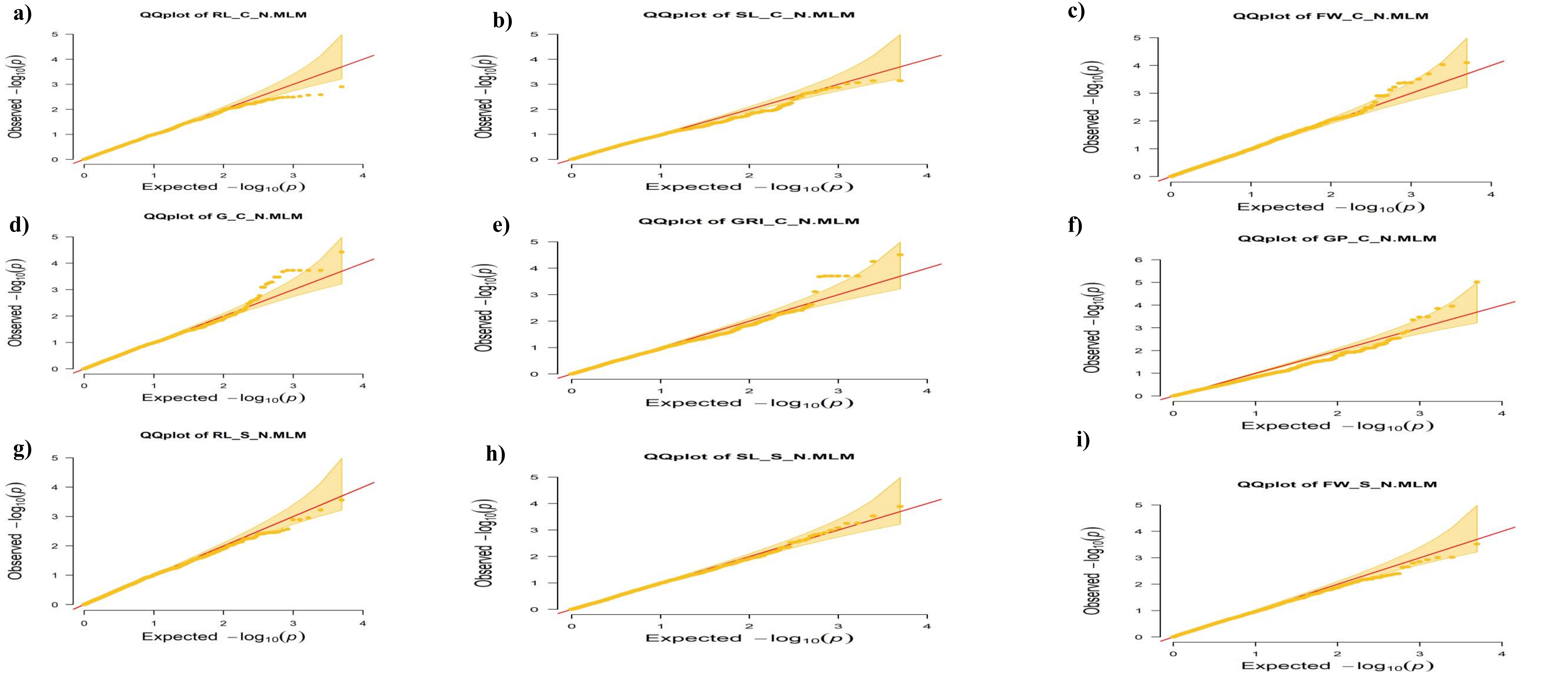

Figure S9: Quantile Quantile plot under control (C), salinity (S), salt tolerance index (STI), and reduction (Red) for nano priming conditions (UP): under control a)Root Length, b)Shoot Length, c)Fresh Weight, d)Germination Percentage, e)Germination Rate Index, and f)Germination Pace ; under salinity g)Root Length, h)Shoot Length, i)Fresh Weight, j)Germination Percentage, k)Germination Rate Index, and l)Germination Pace; for salt tolerance index m)Root Length, n)Shoot Length, o)Fresh Weight, p)Germination Percentage, q)Germination Rate Index, and r)Germination Pace; for reduction s)Root Length, t)Shoot Length, u)Fresh Weight, v)Germination Percentage, w)Germination Rate Index, and x)Germination Pace. The x-axis shows the expected –log10 (P), the y-axis shows the observed -log10 (p).

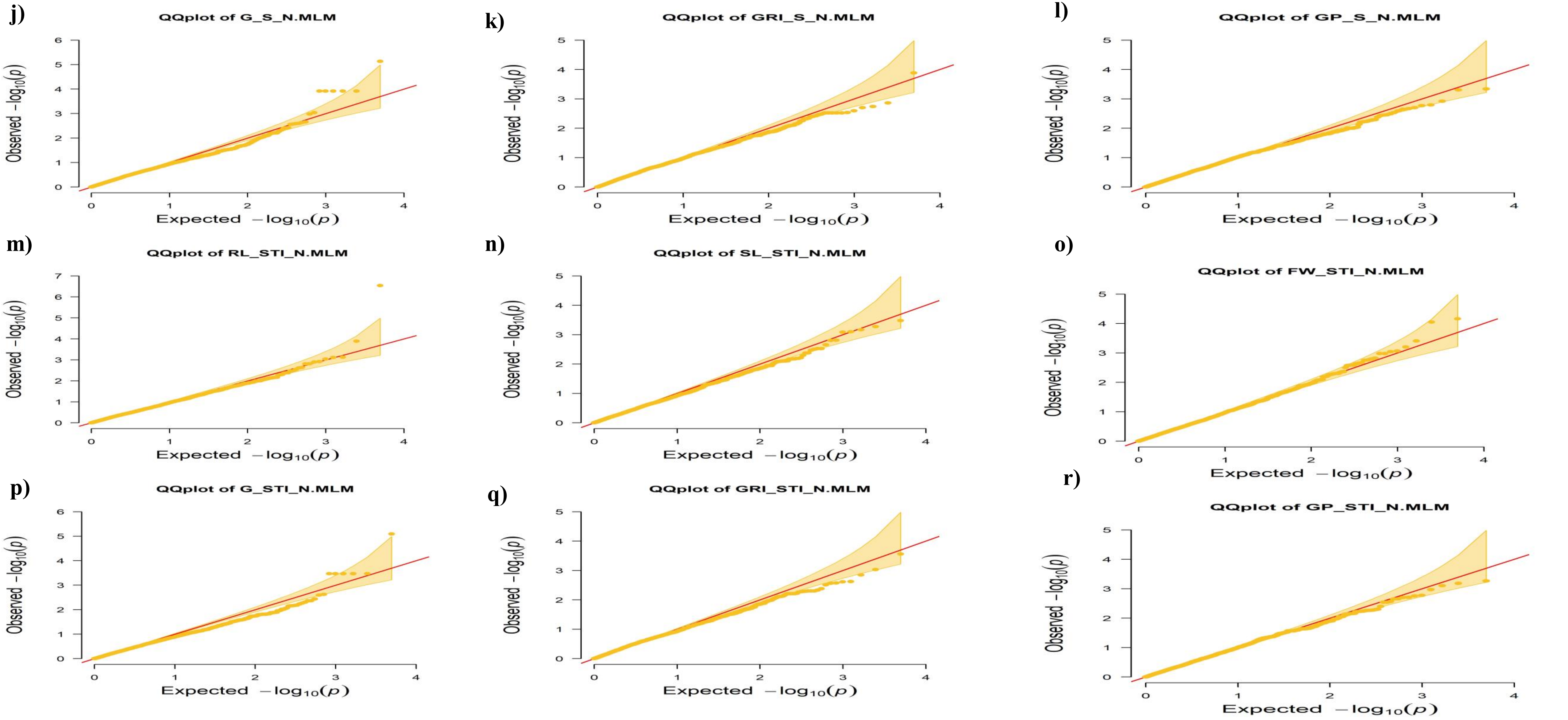

Figure S9 continued from the previous slide : Quantile Quantile plot under control (C), salinity (S), salt tolerance index (STI), and reduction (Red) for nano priming conditions (UP): under control a)Root Length, b)Shoot Length, c)Fresh Weight, d)Germination Percentage, e)Germination Rate Index, and f)Germination Pace ; under salinity g)Root Length, h)Shoot Length, i)Fresh Weight, j)Germination Percentage, k)Germination Rate Index, and l)Germination Pace; for salt tolerance index m)Root Length, n)Shoot Length, o)Fresh Weight, p)Germination Percentage, q)Germination Rate Index, and r)Germination Pace; for reduction s)Root Length, t)Shoot Length, u)Fresh Weight, v)Germination Percentage, w)Germination Rate Index, and x)Germination Pace. The x-axis shows the expected –log10 (P), the y-axis shows the observed -log10 (p)

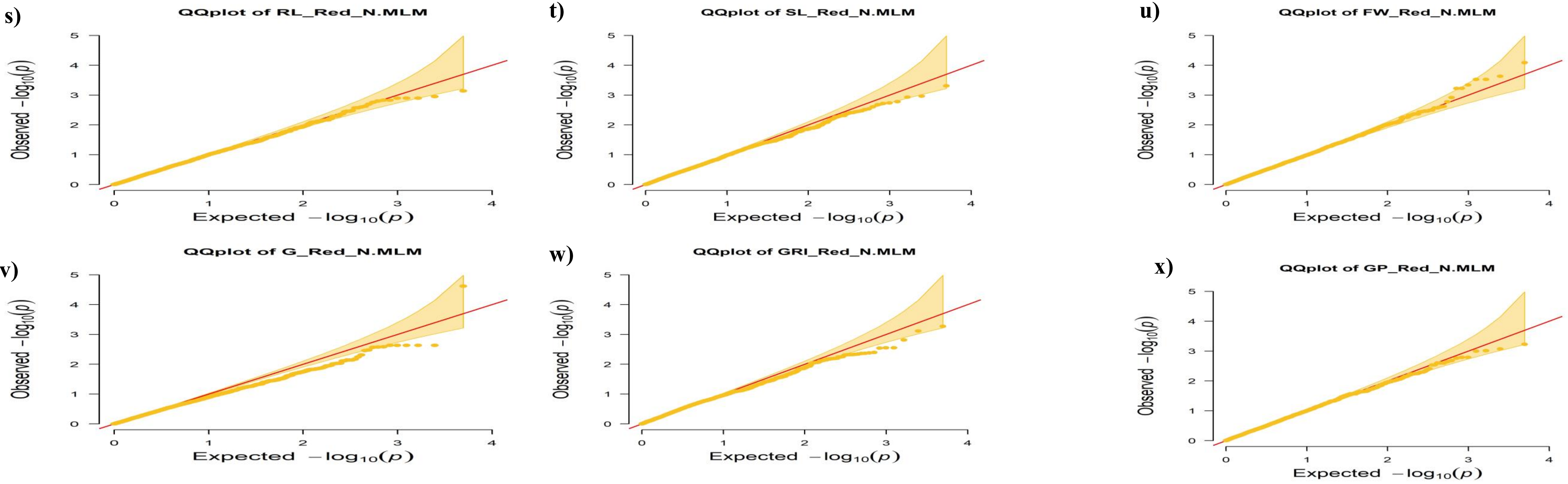

Figure S9 continued from the previous slide : Quantile Quantile plot under control (C), salinity (S), salt tolerance index (STI), and reduction (Red) for nano priming conditions (UP): under control a)Root Length, b)Shoot Length, c)Fresh Weight, d)Germination Percentage, e)Germination Rate Index, and f)Germination Pace ; under salinity g)Root Length, h)Shoot Length, i)Fresh Weight, j)Germination Percentage, k)Germination Rate Index, and l)Germination Pace; for salt tolerance index m)Root Length, n)Shoot Length, o)Fresh Weight, p)Germination Percentage, q)Germination Rate Index, and r)Germination Pace; for reduction s)Root Length, t)Shoot Length, u)Fresh Weight, v)Germination Percentage, w)Germination Rate Index, and x)Germination Pace. The x-axis shows the expected –log10 (P), the y-axis shows the observed -log10 (p).

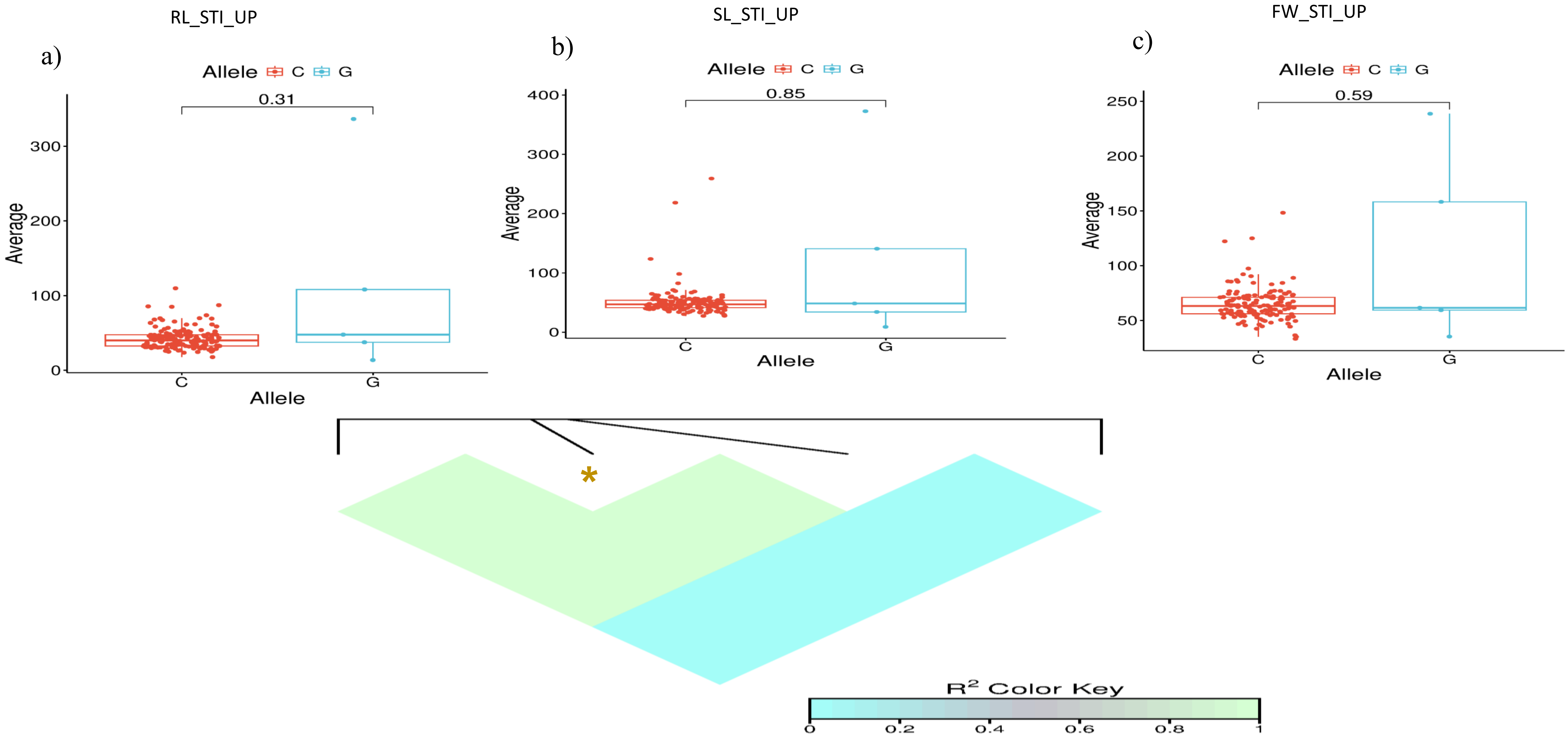

Figure S10: Allelic analysis for the SCRI_RS_227898 which associated with three traits within LD region; a) Root Length_STI_UP, b) Shoot Length_STI_UP, and c) Fresh weight_STI_UP. Where, STI and UP are standing for Salt Tolerance index and Unprimed, respectively. The x-axis shows the allele; the y-axis shows the average of traits.

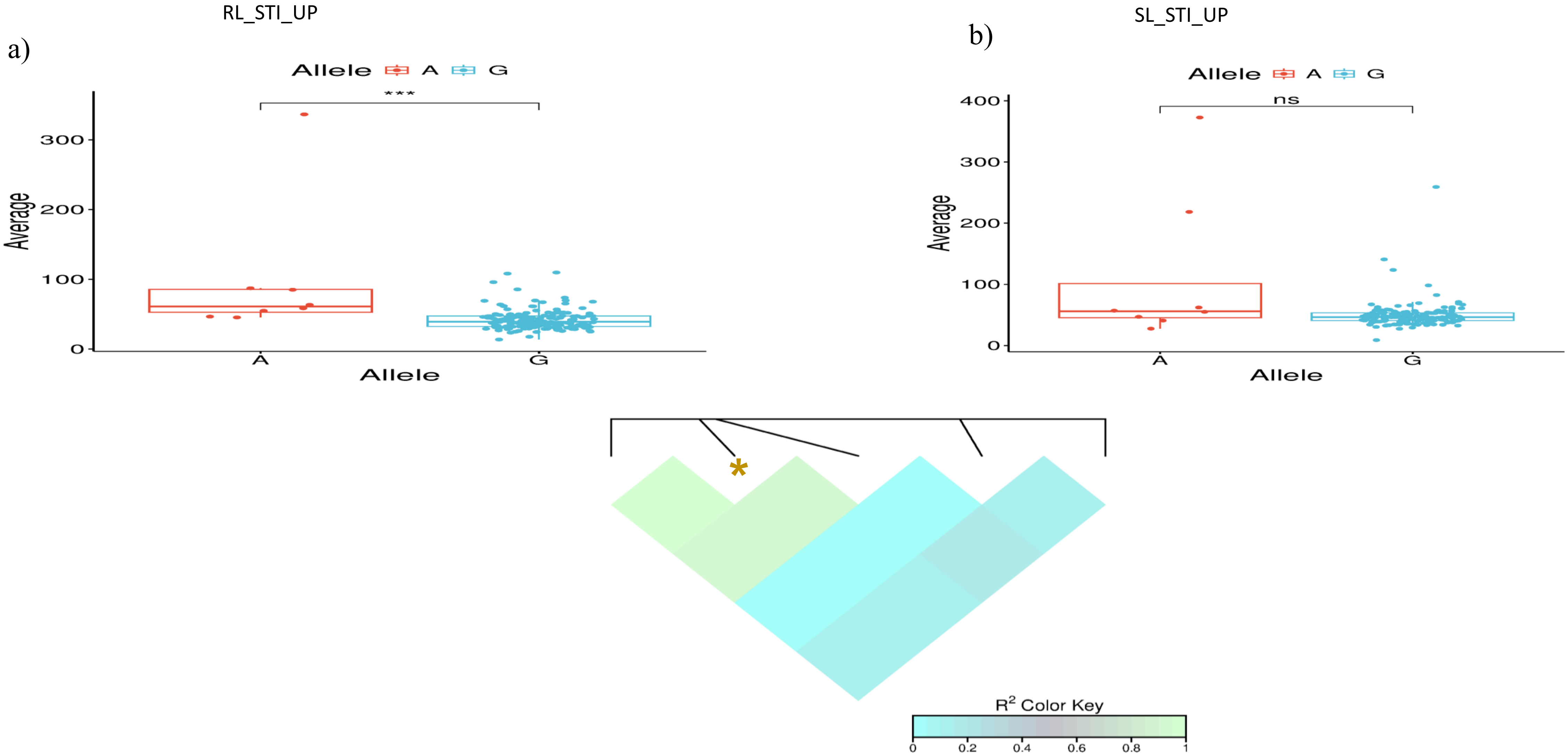

Figure S11: Allelic analysis for the SNP SCRI_RS_168580 which associated with two traits within LD region; a) Root Length_STI_UP and b) Shoot Length_STI_UP. Where, STI, UP, C, and Red are standing for Salt Tolerance index, Unprimed, Control, and Reduction, respectively. The x-axis shows the allele; the y-axis shows the average of traits.
